# Durability of the Single-Dose Ad26.COV2.S Vaccine in the Prevention of COVID-19 Infections and Hospitalizations in the US Before and During the Delta Variant Surge

**DOI:** 10.1001/jamanetworkopen.2022.2959

**Published:** 2022-03-17

**Authors:** Jennifer M. Polinski, Andrew R. Weckstein, Michael Batech, Carly Kabelac, Tripthi Kamath, Raymond Harvey, Sid Jain, Jeremy A. Rassen, Najat Khan, Sebastian Schneeweiss

**Affiliations:** 1 Department of Science, Aetion Inc, New York, New York; 2 Janssen Research and Development Data Science, Spring House, Pennsylvania; 3 Division of Pharmacoepidemiology, Department of Medicine, Brigham and Women’s Hospital and Harvard Medical School, Boston, Massachusetts

## Abstract

**Importance:**

Vaccination against the SARS-CoV-2 virus is critical to control the pandemic. Randomized clinical trials demonstrated efficacy of the single-dose Ad26.COV2.S COVID-19 vaccine, but data on longer-term protection in clinical practice and effectiveness against variants are needed.

**Objective:**

To assess the association between receiving the Ad26.COV2.S vaccine and COVID-19–related infections and hospitalizations before and during the Delta variant surge.

**Design, Setting, and Participants:**

This cohort study included adults aged 18 years and older who were newly Ad26.COV2.S-vaccinated matched to as many as 10 unvaccinated individuals by date, location, age, sex, and comorbidity index. This was followed by 1:4 propensity score matching on COVID-19 risk factors. Data were collected from US insurance claims data from March 1, 2020, through August 31, 2021.

**Exposures:**

Vaccination with Ad26.COV2.S vs no vaccination.

**Main Outcomes and Measures:**

Vaccine effectiveness (VE) was estimated for recorded COVID-19 infection and COVID-19–related hospitalization, nationwide and in subgroups by age, high-risk factors, calendar time, and states with high incidences of the Delta variant. VE estimates were corrected for underrecording of vaccinations in insurance data.

**Results:**

Among 422 034 vaccinated individuals (mean [SD] age, 54.7 [17.4] years; 236 437 [56.0%] women) and 1 645 397 matched unvaccinated individuals (mean [SD] age, 54.5 [17.5] years; 922 937 [56.1%] women), VE was 76% (95% CI, 75%-77%) for COVID-19 infections and 81% (95% CI, 78%-82%) for COVID-19–related hospitalizations. VE was stable for at least 180 days after vaccination and over calendar time. Among states with high Delta variant incidence, VE during June to August 2021 was 74% (95% CI, 71%-77%) for infections and 81% (95% CI, 75%-86%) for hospitalizations. VE for COVID-19 was higher in individuals younger than 65 years (78%; 95% CI, 77%-79%) and lower in immunocompromised patients (64%; 95% CI, 59%-68%). All estimates were corrected for vaccination underrecording; uncorrected VE, which served as a lower bound, was 66% (95% CI, 64%-67%) for any recorded COVID-19 infection and 72% (95% CI, 69%-74%) for COVID-19–related hospitalization.

**Conclusions and Relevance:**

This cohort study in US clinical practice showed stable VE of Ad26.COV2.S for at least 6 months before as well as during the time the Delta variant emerged and became dominant.

## Key Points

**Question:**

What is the durability of the Ad26.COV2.S COVID vaccine effectiveness before and during the Delta variant surge in the US?

**Findings:**

Among 422 034 vaccinated and 1 645 397 matched unvaccinated individuals across the US, vaccine effectiveness was estimated to be 76% for COVID-19 infection and 81% for hospitalizations for at least 180 days after vaccination before and during the Delta variant surge.

**Meaning:**

In this study, the Ad26.COV2.S COVID-19 vaccine was associated with high and durable effectiveness in clinical practice, including against the Delta variant.

## Teaser

This cohort study assesses the association between receiving the Ad26.COV2.S vaccine and COVID-19–related infections and hospitalizations before and during the Delta variant surge.

## Introduction

With the global morbidity and mortality associated with the SARS-CoV-2 virus and the rapid spread of new variants, a temporal understanding of vaccine effectiveness (VE) in clinical practice is critical to inform public health recommendations. On February 27, 2021, the US Food and Drug Administration issued an Emergency Use Authorization for Janssen’s single dose COVID-19 vaccine, Ad26.COV2.S,^1^ based on results from a randomized clinical trial (RCT), ENSEMBLE.^2^ The overall efficacy estimate for preventing moderate to severe COVID-19 at least 14 days after vaccination was 74% in the US (66% globally), and efficacy at least 28 days after vaccination was greater than 84% against severe disease.^3^

Despite the efficacy observed in the RCT, questions remain about generalizability to patients in clinical practice, longer-term effectiveness, and durability amid changes in variant prevalence, such as the Delta variant (B.1.617.2, AY.1, AY.2, and AY.3), which emerged in late May 2021 and became the dominant variant across the US by July.^4^ A recent study^5^ showed the effectiveness of Ad26.COV2.S and other COVID-19 vaccines in US ambulatory and inpatient care settings using data through June 22, 2021. This test-negative study included fewer than 1500 individuals who received the Ad26.COV2.S vaccine and a study period predominantly before the Delta variant became prevalent.^6^ A series of case-control studies by the US Centers for Disease Control and Prevention (CDC) found similar effectiveness as the ENSEMBLE trial in only age-adjusted analyses.^7,8^ A cohort study limited to New York state showed VE estimates of 82% to 85% for COVID-19–related hospitalizations.^9^ A hospital-based case-control study from the Dutch Ministry of Health found 91% VE. Using national claims data, we sought to assess the longer-term stability of the VE of Ad26.COV2.S in preventing COVID-19 infections or COVID-19–related hospitalizations in clinical practice across the US and its durability against variants, including Delta.^10^

## Methods

### Data

We analyzed deidentified patient-level claims data from March 1, 2020, through August 31, 2021, submitted to insurance companies by US providers of inpatient, outpatient, pharmacy, and laboratory services and aggregated by HealthVerity that have been used for other COVID-19–related studies.^11,12,13,14,15^ The claims were anonymously linked across health data sources using a unique, encrypted, and nonidentifiable patient token derived from identifiable information known only to the provider (eMethods 6 in the Supplement). This token allows for cross-database longitudinal capture of diagnosis and procedure codes, COVID-19 laboratory test orders and results, and pharmacy dispensing information, as demonstrated in other studies.^15^ Cohorts for the current study were identified from a larger data set of 168 million individuals with any record of laboratory orders, diagnoses, or treatments broadly related to COVID-19 (COVID-19–related utilization) or COVID-19 vaccinations. This national claims database includes all major insurance types, with proportions representative of the US population: Medicaid (19%), Medicare (14%), commercial (60%), and federal (5%) were balanced and representative of the US population.^16^

This cohort study was approved by the New England institutional review board, and on reasonable request, researchers may get access to the data and analytics infrastructure for prespecified collaborative analyses. The requirement for informed consent was waived owing to the use of deidentified data. The study protocol was originally submitted to the FDA for review in April 2021 and revised in August 2021. We followed the Reporting of Studies Conducted Using Observational Routinely Collected Data (RECORD-PE) reporting guideline.^17^

### Population and Exposure

Individuals aged 18 years or older who received a single dose of Ad26.COV2.S between March 1 and August 17, 2021, entered the study cohort on the day of vaccination. Each participant was matched on the same day to as many as 10 referent individuals with no evidence of a COVID-19 vaccination at that time, with exact matching by location (3-digit zip code), age within 4 years, sex, and general health status captured in a comorbidity score (eMethods 1 in the Supplement).^18^ We required at least 1 medical and pharmacy claim during the 365 days before cohort entry to ensure each individual’s activity in the system. Those with evidence of prior COVID-19 or receipt of any COVID-19 vaccine during the 365 days before cohort entry were excluded (eMethods 2 and eFigure 1 in the Supplement).

### Follow-up and Outcomes

Follow-up in each group started 14 days after cohort entry^19^ and continued until the earliest of the occurrence of an outcome, receipt of any COVID-19 vaccine, death, or August 31, 2021. The outcome of recorded COVID-19 infection was defined by either recording of an inpatient or outpatient *International Classification of Diseases, Ninth Revision, Clinical Modification *(*ICD-10-CM*) diagnosis code of U07.1 (85% of cases) in any position and/or a recorded positive SARS-CoV-2 diagnostic polymerase chain reaction or nucleic acid amplification test result (15%). The coprimary outcome was COVID-19–related hospitalization, defined as any claim for an inpatient stay with a discharge diagnosis of COVID-19 or a recorded infection within 21 days before admission.

### Participant Characteristics

Participant characteristics, including a range of factors associated with severe COVID-19 disease,^2^ were assessed during the 365 days before cohort entry. All definitions appear in eMethods 3 in the Supplement. Comorbidities included chronic obstructive pulmonary disease, asthma, pulmonary fibrosis, cystic fibrosis, HIV, immunocompromised status after transplantation, liver disease, malignant neoplasms, cerebrovascular disease, chronic kidney disease, hypertension, obesity, heart failure, sickle-cell disease, thalassemia, diabetes, chronic neurologic conditions, and an overall comorbidity score.^18^ We measured health care utilization intensity, a marker of patients’ baseline health state and surveillance, including the number of pharmacy or medical claims in the 365 days before cohort entry, and recent medical or pharmacy claims in the 60 days before cohort entry.

### Statistical Analysis

In addition to the 1:10 risk-set sampling exactly matched by day, location, age, sex, and comorbidity score described previously, we further matched participants on a range of pre-exposure risk factors for COVID-19 severity using propensity scores (PSs) to ensure balance between treatment groups. Each vaccinated individual was PS-matched with as many as 4 unvaccinated individuals with a caliper of ±0.01. PSs were estimated with logistic regression including all pre-exposure patient characteristics reported in Table 1, with no further variable selection. The resulting balance of characteristics between the vaccinated and PS-matched unvaccinated individuals was assessed by computing the standardized difference for each covariate.^20^ A value of less than 0.10 indicates little imbalance between treatment groups and little association with residual confounding.^21^

**Table 1.** Characteristics of Individuals Who Received Ad26.COV2.S Vaccination and Matched Unvaccinated Individuals in the National Cohort

Characteristic^a^	Participants, No. (%)		
	Ad26.COV2.S vaccinated (n = 422 034)	Matched unvaccinated (n = 1 645 397)	Absolute standardized difference
Age, mean (SD)	54.65 (17.36)	54.52 (17.47)	0.007
Sex			
Female	236 437 (56.0)	922 937 (56.1)	0.001
Male	185 597 (44.0)	722 460 (43.9)	
Cerebrovascular disease	15 421 (3.7)	60 076 (3.7)	0.000
Chronic kidney disease	21 904 (5.2)	83 640 (5.1)	0.005
Chronic obstructive pulmonary disease	44 479 (10.5)	173 510 (10.5)	0.000
Cystic fibrosis^b^	24 (<0.1)	98 (<0.1)	0.000
HIV	1461 (0.3)	6073 (0.4)	0.004
Hypertension	133 855 (31.7)	517 710 (31.5)	0.005
Immunocompromised state from blood transplant^b^	4 (<0.1)	26 (<0.1)	0.002
Immunocompromised state from organ transplant	1559 (0.4)	6229 (0.4)	0.002
Liver disease	17 970 (4.3)	69 640 (4.2)	0.001
Malignant neoplasms	18 598 (4.4)	72 493 (4.4)	0.000
Moderate-to-severe asthma	3862 (0.9)	15 258 (0.9)	0.001
Neurologic conditions	121 537 (28.8)	472 062 (28.7)	0.002
Obesity	65 458 (15.5)	253 745 (15.4)	0.002
Pulmonary fibrosis	2121 (0.5)	8532 (0.5)	0.002
Serious heart conditions	41 858 (9.9)	163 917 (10.0)	0.001
Sickle-cell disease	195 (<0.1)	895 (0.1)	0.004
Thalassemia	211 (<0.1)	881 (0.1)	0.002
Type 1 diabetes	4546 (1.1)	17 458 (1.1)	0.002
Type 2 diabetes	65 181 (15.4)	250 201 (15.2)	0.007
Gagne combined comorbidity score, mean (SD)	0.65 (1.58)	0.64 (1.56)	0.005
Count of claims, mean (SD)			
Medical	13.48 (35.14)	13.44 (32.73)	0.001
Pharmacy	18.06 (18.96)	17.87 (18.67)	0.010
Recent claim^c^			
Medical	214 715 (50.9)	833 421 (50.7)	0.004
Pharmacy	302 097 (71.6)	1 172 214 (71.2)	0.008
Index months			
March 2021	150 316 (35.6)	584 651 (35.5)	0.004
April 2021	149 472 (35.4)	585 596 (35.6)	
May 2021	73 060 (17.3)	284 240 (17.3)	
June 2021	29 090 (6.9)	113 423 (6.9)	
July 2021	13 674 (3.2)	52 711 (3.2)	
August 2021	6422 (1.5)	24 776 (1.5)	
US Region			
Northeast	56 655 (13.4)	220 319 (13.4)	0.003
Midwest	95 301 (22.6)	370 693 (22.5)	
South	177 041 (41.9)	692 261 (42.1)	
West	93 037 (22.0)	362 124 (22.0)	
State	NA	NA	0.010

Abbreviation: NA, not applicable.

^a^ Characteristics reported for population matched with risk-set sampling and propensity scores. Unless otherwise noted, demographic variables were assessed at cohort entry (index) and comorbidities and clinical utilization variables were assessed during the 1 year before cohort entry.

^b^ Variables excluded from final propensity score models.

^c^ Recent medical and pharmacy claims were defined as claims beginning during the 60 days before cohort entry.

We computed incidence rates per 1000 person-years for vaccinated and unvaccinated participants and hazard ratios (HRs) with 95% CIs.^22^ VE as a percentage was computed as (1 − HR) × 100. Kaplan-Meier curves with 95% CIs and Schoenfeld residuals were plotted to test whether VE changed with time since vaccination.^23^

Additional analyses included stratification by calendar months from March through August 2021 to determine whether there was a consistent VE over calendar time (eMethods 5 and eFigure 3 in the Supplement), including Mann-Kendall trend tests,^24^ as well as stratification by age (<65 and ≥65 years), and clinically relevant subgroups (immunocompromised, HIV, and type 2 diabetes). To examine VE in a time and region where a large fraction of new cases were patients infected with the Delta variant, we separately analyzed data from 4 states with high incidence of the Delta variant in June through August (Florida, Louisiana, Arkansas, and Missouri; hereafter high Delta-incidence states).^25^

Given the expedited national vaccination effort, a sizable proportion of COVID-19 vaccinations were administered by employers, mass vaccination sites, pharmacies, and other settings where often no health insurance claims were submitted. The CDC reported that 60% of US residents 12 years and older were vaccinated as of August 2021, while only 36% of our claims data population had vaccine events recorded. After person-level linkage of HealthVerity claims data with the Louisiana state vaccine registry, we confirmed underrecording of approximately 40% in claims data (eMethods 4 in the Supplement). As a result, it is highly likely that a sizable proportion of patients classified as unvaccinated in our cohort was in fact vaccinated, making our comparison group more protected than a truly unvaccinated group, and thus the observed VE estimates will appear lower than indeed true. To compensate, we assumed 40% underrecording of vaccinations and applied a correction factor to all VE estimates using standard algebraic methods for correcting this exposure misclassification.^26^ In sensitivity analyses, we varied the underrecording assumption from 0% to 70% (eTables 5 and 6 in the Supplement). In addition to corrected estimates, we also report all uncorrected VE estimates as the lower bound of VE within the current study.

We conducted 2 additional sensitivity analyses to confirm the robustness of our analysis. First, we narrowed COVID-19 outcome definitions to require a positive nucleic acid amplification test result for all recorded COVID-19 outcomes (eTable 7 in the Supplement). Second, to minimize potential bias related to differential sampling of vaccinated and unvaccinated individuals at the data extraction stage, we required all individuals to have evidence of potential COVID-19–related utilization before cohort entry (eTable 8 in the Supplement).

All analyses were performed using the Aetion Evidence Platform version 4.33 (including R version 3.4.2 [R Project for Statistical Computing]), which has been scientifically validated by accurately repeating previously published studies^27^ and by replicating^28^ or estimated clinical trial findings.^29^ All transformations of the raw data are preserved for full reproducibility, and audit trails are available, including a quality check of the data ingestion process.

## Results

**Figure 1.**
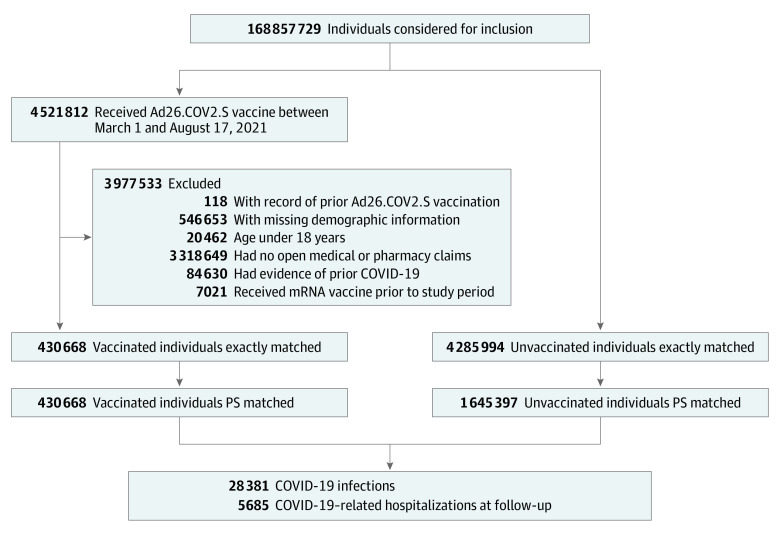
Study Population Flow Diagram Match ratios reflect the maximum number of unvaccinated matches allowed for each vaccinated individual; however, some vaccinated individuals will have fewer than the maximum number of matches. During risk-set sampling, each vaccinated individual was matched with as many as 10 unvaccinated individuals; for propensity score (PS) matching, each vaccinated individual was matched with as many as 4 unvaccinated individuals.

The study cohort included a total of 422 034 vaccinated US individuals (mean [SD] age, 54.7 [17.4] years, 236 437 [56.0%] women) and 1 645 397 matched individuals (mean [SD] age, 54.5 [17.5] years; 922 937 [56.1%] women) with no recording of vaccination from March 1 through August 17, 2021 (Figure 1 and eTable 1 in the Supplement). The 2-stage matching by time, location, sociodemographic variables, and risk factors resulted in highly similar groups with absolute standardized mean differences of 0.01 or less across all characteristics, well below the 0.10 threshold for residual balance (Table 1). In high Delta-incidence states, 32 421 vaccinated individuals were similarly well-balanced when compared with 126 313 unvaccinated individuals, with all absolute standardized differences of 0.03 or less, as were all other patient subgroups (eTables 2 and 3 in the Supplement). Most individuals were followed up for outcomes until the end of the study period (99% of vaccinated and 81% of matched unvaccinated), with 17% of unvaccinated individuals censored on the day they received a COVID-19 vaccine during follow-up. The median duration of follow-up across both groups was 129 days for recorded COVID-19 infections and 130 days for COVID-19–related hospitalization. In the national cohort, 2632 vaccinated (18.6 per 1000 person-years) and 25 749 unvaccinated individuals (53.5 per 1000 person-years) had a recorded COVID-19 infection (Table 2). The VE for recorded COVID-19 infection was 76% (95% CI, 75%-77%). Incidence rates of COVID-19–related hospitalization were 3.1 per 1000 person-years in vaccinated individuals and 10.8 per 1000 person-years in unvaccinated individuals. resulting in a corrected VE of 81% (95% CI, 78%-82%) for COVID-19–related hospitalization. The VE for recorded COVID-19 infection was higher in patients younger than 65 years (78%; 95% CI, 77%-79%) than in patients 65 years or older (72%; 95% CI, 70%-74%) (eFigure 4 in the Supplement). Immunocompromised individuals had lower VE (64%; 95% CI, 59%-68%) compared with nonimmunocompromised individuals (77%; 95% CI, 76%-78%), and individuals with type 2 diabetes had lower VE (67%; 95% CI, 64%-70%) compared with individuals without type 2 diabetes (78%; 95% CI, 77%-79%) (eFigure 4 in the Supplement). VE estimates within the HIV subgroup were difficult to interpret due to low outcome counts and wide confidence intervals (Table 2). Uncorrected VE, which served as a lower bound, was 66% (95% CI, 64%-67%) for any recorded COVID-19 infection and 72% (95% CI, 69%-74%) for COVID-19–related hospitalization.

**Table 2.** Incidence of COVID-19 and COVID-19–Related Hospitalizations and Vaccine Effectiveness

Subgroup (No. of vaccinated vs No. of unvaccinated)	Ad26.COV2.S vaccinated group			Matched unvaccinated group			Observed hazard ratios (95% CI)	Vaccine effectiveness, % (95% CI)	
	Events. No.	Person-years	Incidence rate, per 1000 person-years	Events, No.	Person-years	Incidence rate, per 1000 person-years		Observed	Corrected for vaccine underrecording^a^
National cohort (422 034 vs 1 645 397)									
Any recorded COVID-19	2632	141 717	18.57	25 749	481 083	53.52	0.34 (0.33-0.36)	66 (64-67)	76 (75-77)
COVID-19–related hospitalization	440	142 047	3.10	5245	484 198	10.83	0.28 (0.26-0.31)	72 (69-74)	81 (78-82)
High Delta-incidence states (32 421 vs 126 313)^b^									
Recorded COVID-19									
Any	372	10 691	34.80	3466	36 564	94.79	0.36 (0.32-0.40)	64 (60-68)	75 (72-78)
June to August 2021 only	327	7399	44.2	2948	24 814	118.8	IRR, 0.37 (95% CI, 0.33-0.41)	63 (59-67)	74 (71-77)
COVID-19–related hospitalization									
Any	61	10 726	5.69	718	36 910	19.45	0.29 (0.22-0.37)	71 (63-78)	80 (75-85)
June to August 2021 only	49	7431	6.59	592	25 127	23.56	IRR, 0.28 (95% CI, 0.21-0.37)	72 (63-79)	81 (75-86)
Subgroups within national cohort									
Age <65 (296 632 vs 1 156 090)									
Any recorded COVID-19	1880	97 790	19.22	19 155	325 147	58.91	0.32 (0.31-0.34)	68 (66-69)	78 (77-79)
COVID-19–related hospitalization	188	98 044	1.92	2862	327 629	8.74	0.22 (0.19-0.25)	78 (75-81)	85 (83-87)
Age ≥65 (125 402 vs 488 902)									
Any recorded COVID-19	752	43 927	17.12	6722	155 775	43.15	0.39 (0.37-0.42)	61 (58-63)	72 (70-74)
COVID-19–related hospitalization	252	44 004	5.73	2350	156 437	15.02	0.38 (0.33-0.43)	62 (57-67)	74 (70-77)
Immunocompromised (29 115 vs 114 325)									
Any recorded COVID-19	246	9915	24.81	1753	34 969	50.13	0.49 (0.43-0.56)	51 (44-57)	64 (59-68)
COVID-19–related hospitalization	68	9946	6.84	519	35 183	14.75	0.46 (0.36-0.60)	54 (40-64)	67 (57-74)
Not immunocompromised (392 919 vs 1 530 417)									
Any recorded COVID-19	2386	131 802	18.10	23 971	446 039	53.74	0.33 (0.32-0.35)	67 (65-68)	77 (76-78)
COVID-19–related hospitalization	372	132 101	2.82	4603	448 944	10.25	0.27 (0.25-0.30)	73 (70-75)	82 (80-83)
HIV positive (1710 vs 6735)									
Any recorded COVID-19	14	576	24.33	68	2072	32.82	0.74 (0.42-1.32)	26 (−32-58)	NA
COVID-19–related hospitalization	4	578	6.92	26	2079	12.51	0.56 (0.20-1.61)	44 (−61-80)	NA
Not HIV positive (420 324 vs 1 638 342)									
Any recorded COVID-19	2618	141 142	18.55	25 808	479 065	53.87	0.34 (0.33-0.36)	66 (64-67)	76 (75-77)
COVID-19–related hospitalization	436	141 470	3.08	5206	482 186	10.80	0.28 (0.26-0.31)	72 (69-74)	81 (79-82)
Type 2 diabetes (65 342 vs 254 739)									
Any recorded COVID-19	592	22 417	26.41	4524	76 702	58.98	0.45 (0.41-0.49)	55 (51-59)	67 (64-70)
COVID-19–related hospitalization	159	22 483	7.07	1524	77 182	19.75	0.36 (0.30-0.42)	64 (58-70)	74 (70-79)
No type 2 diabetes (356 692 vs 1 389 765)									
Any recorded COVID-19	2040	119 300	17.10	21 234	404 307	52.52	0.32 (0.31-0.34)	68 (66-69)	78 (77-79)
COVID-19–related hospitalization	281	119 564	2.35	3643	406 970	8.95	0.26 (0.23-0.30)	74 (70-77)	82 (80-85)

Abbreviations: IRR, incidence rate ratio; NA, not applicable.

^a^ Corrected vaccine effectiveness estimates were adjusted for underrecording of vaccinations in claims data using the approach described in eMethods 4 in the Supplement, assuming 40% underrecording of vaccinations in claims data. There were insufficient outcome counts for application of undercorrection methods within the HIV-positive subgroup.

^b^ High Delta-incidence states (early Delta states) include Arkansas, Florida, Louisiana, and Missouri. For June to August 2021 results within those 4 states, IRRs after propensity score matching are reported instead of hazard ratios, and vaccine effectiveness was estimated using (1 − IRR) × 100 for patients contributing follow-up time from June 1 through August 31, 2021.

In high Delta-incidence states, rates of recorded COVID-19 infections were higher in both groups than in the national cohort, with incidences of 34.8 per 1000 person-years for vaccinated individuals vs 94.8 per 1000 person-years for unvaccinated individuals (Table 2). In these states, the VE for recorded COVID-19 infections was 75% (95% CI, 72%-78%) overall and 74% (95% CI, 71%-77%) during June through August (Table 2), when Delta variant incidence was highest; for COVID-19–related hospitalization, the VE was 80% (95% CI, 75%-85%) overall and 81% (95% CI, 75%-86%) during June through August. VE within subgroups of the high Delta-incidence states for both outcomes was similar to the national cohort (eTable 4 in the Supplement).

**Figure 2.**
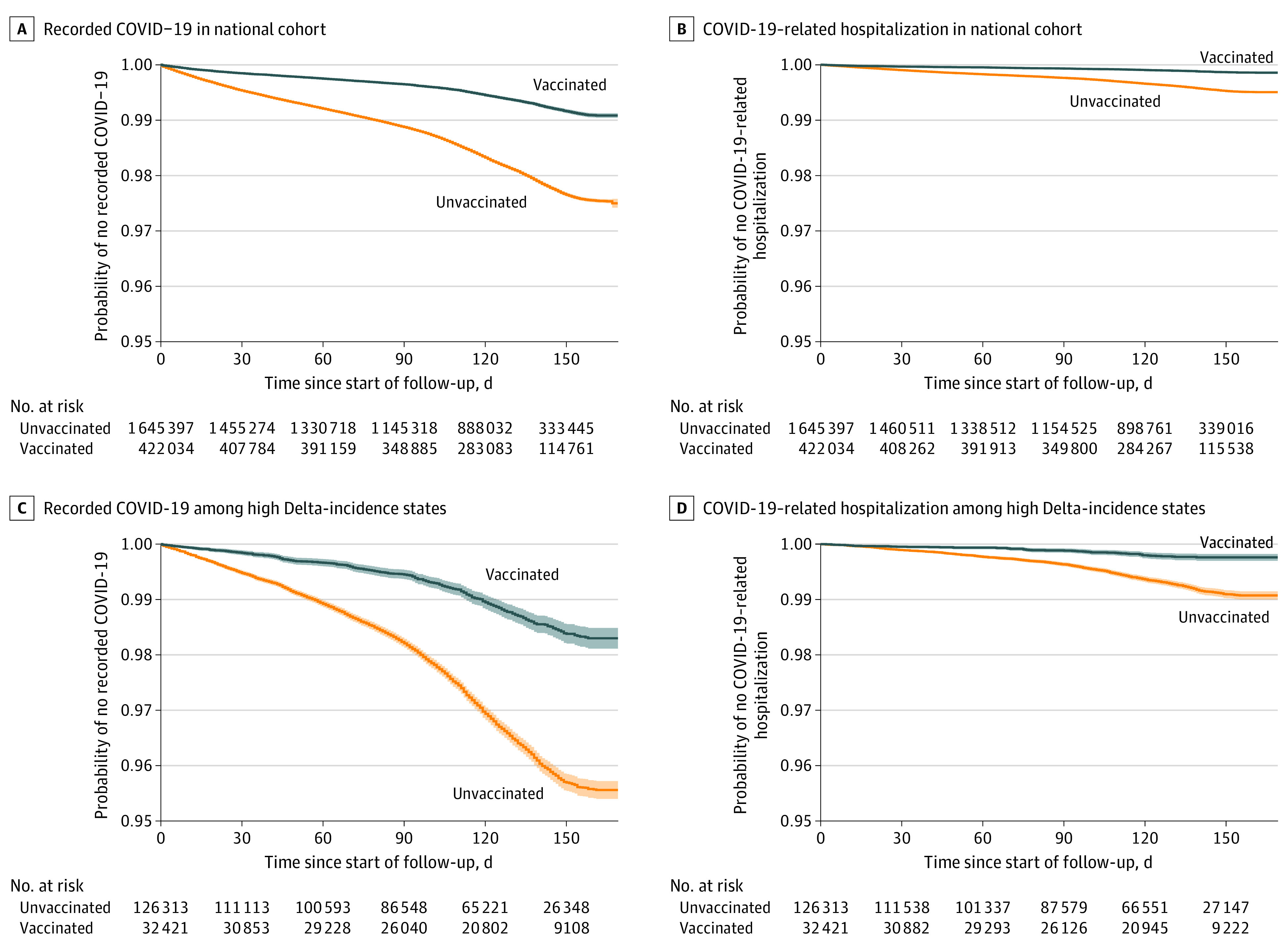
COVID-19–Related Outcomes by Time Since Vaccination Follow-up day 0 is equivalent to 14 days after the index date, as follow-up for outcomes started 14 days after vaccination or matched index date. Kaplan-Meier plots include 95% CIs around the survival function, indicated by shaded areas. Inspection of Schoenfeld residual plots for each outcome indicate that hazard ratios were constant over time and that there were no vaccine effectiveness reductions during observable follow-up time (eFigure 2 in the Supplement). Kaplan-Meier survival curves are based on uncorrected (ie, observed) data.

**Figure 3.**
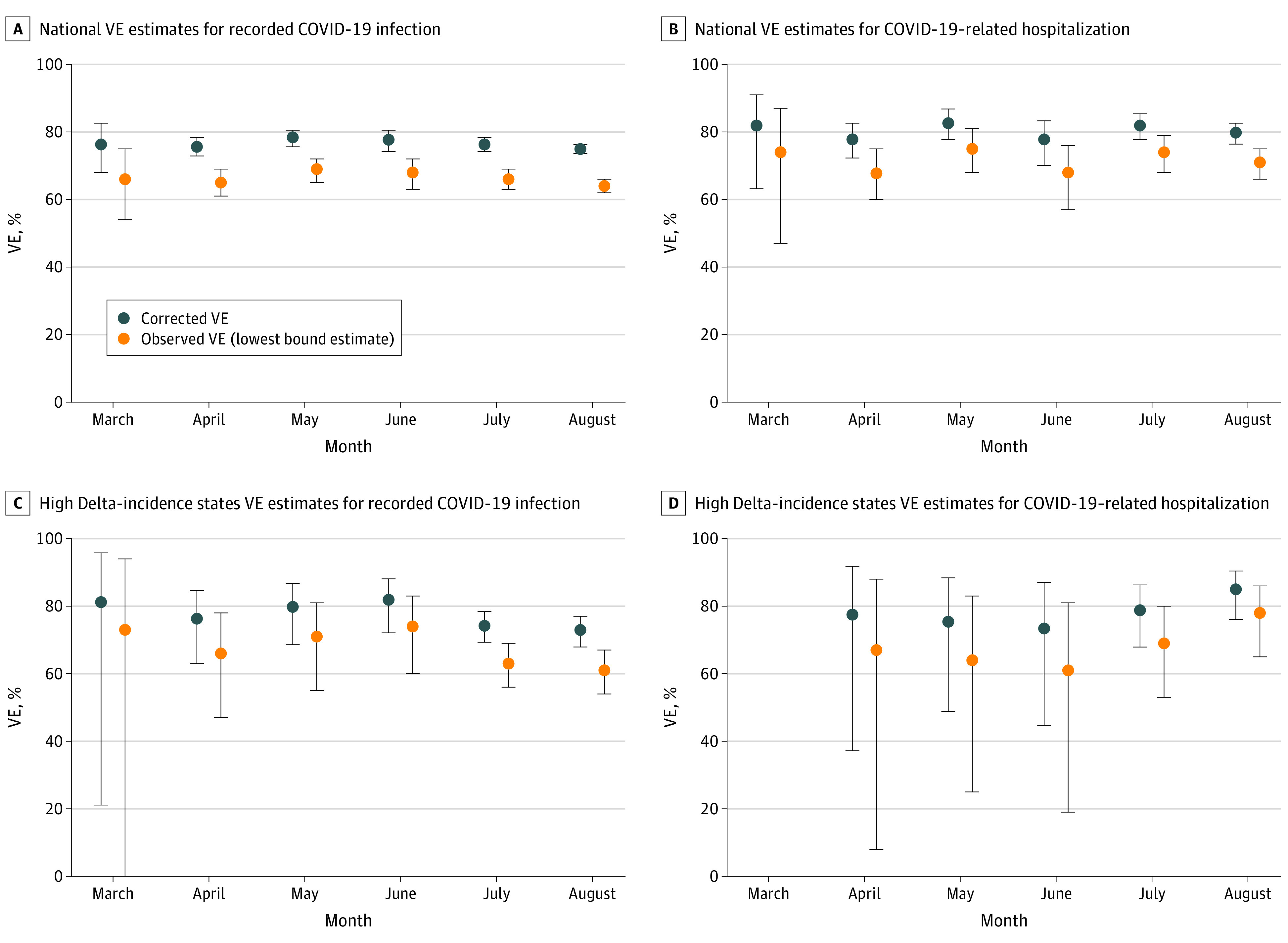
Vaccine Effectiveness (VE) by Month, March Through August 2021 eMethods 5 and eFigure 3 in the Supplement have further detail on methods used to calculate monthly VE estimates. For both outcomes in the national and high Delta-incidence cohorts, all 2-sided *P* values from the Mann-Kendall monotonic trend test were greater than .25, indicating no meaningful change in VE over calendar months (ie, a nonzero slope). Corrected estimates are corrected for underrecording, as described in eMethods 4 in the Supplement, assuming 40% underrecording of vaccinations in claims data. D, There was insufficient outcome count for March-specific effectiveness calculations.

In an analysis of the incidence of recorded COVID-19 infections and COVID-19–related hospitalization as a function of time since vaccination, we observed sustained and stable VE starting 14 days after vaccination to a maximum of 183 days after vaccination for both the national cohort and high Delta-incidence states (Figure 2). Schoenfeld residual plots confirmed that HRs were constant from time since vaccination through the entire observable follow-up period (eFigure 2 in the Supplement). An analysis by calendar months from March through August 2021 showed no meaningful variation in VE over calendar time before and during the Delta variant surge (Mann-Kendall trend tests, *P* > .25). The VE for recorded COVID-19 infections remained stable until the end of the follow-up period in August (75%; 95% CI, 74%-76%), when the Delta variant had spread widely across the US (Figure 3A). The monthly VE estimates for COVID-19–related hospitalization were equally stable (Figure 3B). In the high Delta-incidence states we observed stable VE for both outcomes from March to June, followed by a slight decrease in VE for recorded COVID-19 infections (Figure 3C) and increased fluctuations in VE for hospitalizations (Figure 3D) given smaller event numbers (eTables 8 and 9 in the Supplement). Subgroup analyses followed similar patterns as the time since vaccination analyses (eFigures 5 and 6 in the Supplement).

Results in a sensitivity analysis defining COVID-19 infections by requiring nucleic acid amplification test results for all outcomes were similar to primary findings for the recorded COVID-19 infection outcome defined with laboratory results or diagnosis codes (eTable 7 in the Supplement). When we required all participants to have had COVID-19–related health care encounters before cohort entry, the corrected VE was 54% (95% CI, 52%-56%) for COVID-19 and 63% (95% CI, 58%-68%) for COVID-19–related hospitalization (eTable 8 in the Supplement).

## Discussion

In US clinical practice from March through August 2021, the single-dose Ad26.COV2.S vaccine showed effective protection against recorded COVID-19 and COVID-19–related hospitalization that was stable for at least 180 days after a single vaccination and durable during the surge of the Delta variant. In states with early emergence and high incidence of the Delta variant, we found equally high and sustained VE. These results suggest that the VE observed in the ENSEMBLE trial translate into clinical practice, last for at least 6 months after vaccination, and remain effective amid high Delta variant incidence. This is compatible with long-term immunogenicity data and long-term follow-up of the ENSEMBLE trial participants.^30^

VE remained stable and durable in multiple subgroups, although at varying levels of VE. Individuals 65 years and older as well as immunocompromised patients who were vaccinated with a single dose had slightly reduced yet still substantial protection against COVID-19 and COVID-19–related hospitalizations, which is consistent with the age-dependent variation in VE observed in the ENSEMBLE trial.^3^

Serological studies indicate similar durability of immunological response.^31^ A recent durability study across 3 available vaccines using US national claims data showed equally stable VE regarding COVID-19 hospitalization and minor waning for COVID-19 infections among those vaccinated with Ad26.COV2.S and substantially more waning for the mRNA vaccines.^32^

This national cohort study has several distinct advantages. First, compared with regional US-based cohort analyses^9^ or international studies,^33,34^ it is representative of the US population across all regions with proportional representation of Medicare and Medicaid beneficiaries in addition to commercially and federally insured individuals. Second, as a longitudinal cohort study, it is better-suited than case-control sampling designs^5,6,7,8^ to study whether and when a vaccine begins waning and how VE is affected by changes in variant prevalence by time and location. The alternative case-negative study design is further subject to control sampling issues and false-negative test results, while a cohort study focusing on clinically manifest cases is not.^35,36^ Third, our cohort study focused on clinically symptomatic disease that required a physician visit with or without COVID-19 test results. This makes our study more focused on clinically moderate to severe disease to inform clinical practice. Fourth, our study applied exact matching followed by PS matching to balance participants in risk factors for virus circulation and susceptibility, including time, location, age, sex, and insurance status, and in 17 risk factors for disease severity. While this cannot guarantee complete control for confounding, it makes residual confounding unlikely to have a meaningful impact, particularly given that the pre-Delta findings are closely aligned with existing RCT data. We are not aware of other cohort or case-control studies that applied this level of methodological rigor to overcome the lack of baseline confounding. Unlike RCTs, the timeliness at which these analyses can be performed in national data—and updated as external circumstances change—address an otherwise difficult to fill public health evidence gap that considers the changing prevalence of variants, variations in government mandates, individual behavior, and other quickly evolving factors.

### Limitations

This study has limitations. It is mainly based on the data that flow from US health care professionals to insurance companies for reimbursement and are available sooner than traditional adjudicated (ie, closed) insurance claims. While other studies have used these open claims data to study COVID-19,^12^ the data include no enrollment dates in specific insurance plans. Our study therefore required the occurrence of clinician-based medical and pharmacy claims unrelated to COVID-19 during baseline to restrict our population to those with most complete outcome capture, making it unlikely that there is differential capture of information between the groups. Although this necessary restriction to ensure high data capture restricts the study population, it remained highly representative of the US insured population (eMethods 7 in the Supplement). Even if it would result in underrecording of baseline factors or events, the longitudinal nature of our study and conclusions of stable VE since vaccination and lack of substantial month-to-month changes in VE are unlikely to be affected by this.

As a result of mass vaccination efforts by a range of public and private organizations, where health insurance information may not have been required, collected, or submitted, a substantial fraction of COVID-19 vaccine administrations were not recorded in insurance data.^37^ Given the underrecording of COVID-19 vaccinations in insurance claims data, which leads to an underestimation of the true effect size, we empirically estimated the level of underrecording by calibrating against national and state vaccine registry data (eMethods 4 in the Supplement) and applied standard correction methods to all VE estimates.^38^ Even if one would falsely assume no (0%) underrecording and completely disregard the necessary correction, the VE estimates would decline by about 10 percentage points, as illustrated in Table 2, eTable 5, and eTable 6 in the Supplement. The resulting biased and underestimated VE of 66% (95% CI, 64%-67%) for infections and 72% (95% CI, 69%-74%) for hospitalizations demonstrates substantial and durable vaccine protection.

Recent seroprevalence data indicate a meaningful proportion of undocumented infections.^39^ Such nondifferentially reduced sensitivity to capture nonsymptomatic infections would lead to an underestimation of the reported VE on recorded COVID-19 infections but would not affect the VE for COVID-19–related hospitalizations.^38^ Additionally, COVID-19 outcomes captured with *ICD-10* diagnosis codes in claims data may indicate more severe cases requiring physician consultation as opposed to individuals with asymptomatic or mild to moderate disease who may be less likely to seek professional care and generate claims.^40,41^ While it is unclear how exactly the infection severity definition compares with definitions used in RCTs, we suggest comparing our findings with the RCT’s severe or moderate to severe definition.^2^ Consistency of VE results across sensitivity analyses with laboratory-only outcome definitions provides further evidence for the robustness of our primary results (eTable 7 in the Supplement).

Although we observed a high VE in the US through August 2021, when the Delta variant was dominant per CDC sequence data (>90% prevalence),^10^ we did not have sequence-specific information in our data. Hence, we do not know with certainty the Delta variant–specific VE. The Delta variant circulated substantially in the US through September 2021, and this analysis included data through the end of August, covering the majority impact of Delta. HIV and immunocompromised subgroups were defined at baseline and thus did not consider whether individuals were receiving antiretroviral or immunosuppressant therapies during the outcome observation period.

## Conclusions

This population-based cohort study from clinical practice in the US found that Ad26.COV2.S was associated with stable and durable VE for at least 6 months across high-risk patient subgroups and in geographic areas with high Delta variant incidence. As the COVID-19 pandemic continues and variants evolve, the observed VE may change as new variants emerge. Recent data have shown that a second dose will further improve protection.^42^ Our data and data to be generated repeatedly over the coming months using this system of claims data^43^ contribute important and otherwise unattainable evidence specific to policy makers, physicians, and residents of the US.

## Notes

Supplement. **eMethods 1.** Risk-Set Sampling of Unvaccinated Study Individuals **eMethods 2.** Codes Used to Identify Cohort Inclusion and Exclusion Variables **eMethods 3.** Codes of Pre-exposure Covariates Used for Confounding Adjustment via Propensity Score Matching **eMethods 4.** Vaccine Underreporting and Correction **eMethods 5.** Month-by-Month Effectiveness Analyses **eMethods 6.** HealthVerity Data Linkage Overview **eMethods 7.** Representativeness of Study Population as Compared With US Census Bureau Estimates
eReferences.
**eFigure 1.** Study Design Diagram **eFigure 2.** Schoenfeld Residuals Plot for Primary Analysis in National Cohort and High Delta-Incidence States **eFigure 3.** Diagrams for Methods Used to Calculate Month-Specific Effectiveness Estimates **eFigure 4.** COVID-19–Related Outcomes by Time Since Vaccination (+14 Days) for Subgroups Within National Cohort (Kaplan-Meier Plots) **eFigure 5.** Month-by-Month VE Plots for Subgroups Within National Cohort **eFigure 6.** Plot of Schoenfeld Residuals for Subgroups Within National Cohort **eTable 1.** Study Population Counts and Attrition **eTable 2.** Characteristics of Ad26.COV2.S Vaccinated and Matched Unvaccinated Individuals in National Cohort, by Subgroup **eTable 3.** Characteristics of Ad26.COV2.S Vaccinated and Matched Unvaccinated Individuals in High Delta-Incidence States, Overall and by Subgroup **eTable 4.** Incidence and Vaccine Effectiveness for COVID-19 and COVID-19–Related Hospitalizations: High Delta-Incidence States, Overall and by Subgroup **eTable 5.** Correction of the VE Regarding Any Recorded COVID-19 Infection With Varying Assumptions About the Level of Underrecording in Claims Data **eTable 6.** Correction of the VE Regarding COVID-19–Related Hospitalizations With Varying Assumptions About the Level of Underrecording in Claims Data **eTable 7.** Incidence and Vaccine Effectiveness for COVID-19 and COVID-19–Related Hospitalizations Defined by Laboratory NAAT Results Only **eTable 8.** Incidence and Vaccine Effectiveness for COVID-19 and COVID-19–Related Hospitalizations Among Cohort Meeting Data Extraction Criteria Before Cohort Entry **eTable 9.** Characteristics of Ad26.COV2.S Vaccinated and Risk-Set Sample Matched Unvaccinated Individuals in National Cohort Prior to Propensity Score Matching
