# Supplementary material for: Durability of the Single-Dose Ad26.COV2.S Vaccine in the Prevention of COVID-19 Infections and Hospitalizations in the US Before and During the Delta Variant Surge
Source: JAMA Netw Open. 2022 Mar 17;5(3):e222959. doi: 10.1001/jamanetworkopen.2022.2959 (PMC8931562; doi:10.1001/jamanetworkopen.2022.2959)
Supplement: Supplement. — eMethods 1. Risk-Set Sampling of Unvaccinated Study Individuals eMethods 2. Codes Used to Identify Cohort Inclusion and Exclusion Variables eMethods 3. Codes of Pre-exposure Covariates Used for Confounding Adjustment via Propensity Score Matching eMethods 4. Vaccine Underreporting and Correction eMethods 5. Month-by-Month Effectiveness Analyses eMethods 6. HealthVerity Data Linkage Overview eMethods 7. Representativeness of Study Population as Compared With US Census Bureau Estimates eReferences. eFigure 1. Study Design Diagram eFigure 2. Schoenfeld Residuals Plot for Primary Analysis in National Cohort and High Delta-Incidence States eFigure 3. Diagrams for Methods Used to Calculate Month-Specific Effectiveness Estimates eFigure 4. COVID-19–Related Outcomes by Time Since Vaccination (+14 Days) for Subgroups Within National Cohort (Kaplan-Meier Plots) eFigure 5. Month-by-Month VE Plots for Subgroups Within National Cohort eFigure 6. Plot of Schoenfeld Residuals for Subgroups Within National Cohort eTable 1. Study Population Counts and Attrition eTable 2. Characteristics of Ad26.COV2.S Vaccinated and Matched Unvaccinated Individuals in National Cohort, by Subgroup eTable 3. Characteristics of Ad26.COV2.S Vaccinated and Matched Unvaccinated Individuals in High Delta-Incidence States, Overall and by Subgroup eTable 4. Incidence and Vaccine Effectiveness for COVID-19 and COVID-19–Related Hospitalizations: High Delta-Incidence States, Overall and by Subgroup eTable 5. Correction of the VE Regarding Any Recorded COVID-19 Infection With Varying Assumptions About the Level of Underrecording in Claims Data eTable 6. Correction of the VE Regarding COVID-19–Related Hospitalizations With Varying Assumptions About the Level of Underrecording in Claims Data eTable 7. Incidence and Vaccine Effectiveness for COVID-19 and COVID-19–Related Hospitalizations Defined by Laboratory NAAT Results Only eTable 8. Incidence and Vaccine Effectiveness for COVID-19 and COVID-19–Related Hospitali [file jamanetwopen-e222959-s001.pdf]

Supplemental Online Content

Polinski JM, Weckstein AR, Batech M, et al. Durability of the single-dose Ad26.COV2.S vaccine in the prevention of COVID-19 infections and hospitalizations in the US before and during the Delta variant surge. *JAMA Netw Open.* 2022;5(3):e222959. doi:10.1001/jamanetworkopen.2022.2959

eMethods 1. Risk-Set Sampling of Unvaccinated Study Individuals

eMethods 2. Codes Used to Identify Cohort Inclusion and Exclusion Variables

eMethods 3. Codes of Pre-exposure Covariates Used for Confounding Adjustment via Propensity Score Matching

eMethods 4. Vaccine Underreporting and Correction

eMethods 5. Month-by-Month Effectiveness Analyses

eMethods 6. HealthVerity Data Linkage Overview

eMethods 7. Representativeness of Study Population as Compared With US Census Bureau Estimates

eReferences.

eFigure 1. Study Design Diagram

eFigure 2. Schoenfeld Residuals Plot for Primary Analysis in National Cohort and High Delta-Incidence States

eFigure 3. Diagrams for Methods Used to Calculate Month-Specific Effectiveness Estimates

eFigure 4. COVID-19–Related Outcomes by Time Since Vaccination (+14 Days) for Subgroups Within National Cohort (Kaplan-Meier Plots)

eFigure 5. Month-by-Month VE Plots for Subgroups Within National Cohort

eFigure 6. Plot of Schoenfeld Residuals for Subgroups Within National Cohort

eTable 1. Study Population Counts and Attrition

eTable 2. Characteristics of Ad26.COV2.S Vaccinated and Matched Unvaccinated Individuals in National Cohort, by Subgroup

eTable 3. Characteristics of Ad26.COV2.S Vaccinated and Matched Unvaccinated Individuals in High Delta-Incidence States, Overall and by Subgroup

eTable 4. Incidence and Vaccine Effectiveness for COVID-19 and COVID-19–Related Hospitalizations: High Delta-Incidence States, Overall and by Subgroup

eTable 5. Correction of the VE Regarding Any Recorded COVID-19 Infection With Varying Assumptions About the Level of Underrecording in Claims Data

eTable 6. Correction of the VE Regarding COVID-19–Related Hospitalizations With Varying Assumptions About the Level of Underrecording in Claims Data

© 2022 Polinski JM et al. *JAMA Network Open.*

eTable 7. Incidence and Vaccine Effectiveness for COVID-19 and COVID-19–Related Hospitalizations Defined by Laboratory NAAT Test Results Only

eTable 8. Incidence and Vaccine Effectiveness for COVID-19 and COVID-19–Related Hospitalizations Among Cohort Meeting Data Extraction Criteria Before Cohort Entry

eTable 9. Characteristics of Ad26.COV2.S Vaccinated and Risk-Set Sample Matched Unvaccinated Individuals in National Cohort Prior to Propensity Score Matching

This supplemental material has been provided by the authors to give readers additional information about their work.

© 2022 Polinski JM et al. *JAMA Network Open.*

eMethods 1. Risk-Set Sampling of Unvaccinated Study Individuals

A two-step process was used to identify a referent group of non-vaccinated individuals available for follow-up on the same date and geography as all identified exposed individuals. This two-step process first included risk-set sampling via exact matching exposed (vaccinated) individuals with up to ten non-vaccinated (referent) individuals on: time (calendar date), age (within age categories of 18-24; 25-29; 30-34; 35-39; 40-44; 45-49; 50-54; 55-59; 60-64; 65-69; 70-74; 75-79; 80-84; 85+), sex, geography (based on 3-digit ZIP codes), and Gagne Combined Comorbidity Index based on 365 day baseline period (categories: 0-1, 2-3, 4-5, 6+).

From these risk-set sampled patients, PS matching as described in the manuscript was performed to obtain final matched cohorts.

© 2022 Polinski JM et al. *JAMA Network Open.*

eMethods 2. Codes Used to Identify Cohort Inclusion and Exclusion Variables

Variable name	RWD Definition
Administration of the Janssen Ad26.COV2.S vaccine (inclusion)	Any medical claim, pharmacy claim, inpatient hospital encounter, or outpatient hospital encounter with one of the following CPT or NDC codes recorded:<br>CPT codes: 91303, 0031A<br>NDC codes: 59676-580-05, 59676-0580-05, 59676-0580-15, 59676-580-15<br>OR<br>Any retail pharmacy claim for a COVID-19 vaccine with the following manufacturer name: "JANSSEN", "JANSSEN (DIVISION OF JOHNSON AND JOHNSON)", "JOHNSON AND JOHNSON", "JSN" and CVX code is 212<br><br>Note: CVX codes are codes that indicate the product used in a vaccination and are maintained by the CDC's National Center of Immunization and Respiratory Diseases.
Evidence of prior observed COVID-19 (exclusion)	Any medical claim, inpatient hospital encounter, or outpatient hospital encounter with one of the following ICD-10-CM codes:<br>U07.1<br>Z20.822<br>Z86.16<br>J12.82<br>OR<br>Record of any positive result from a COVID-19 diagnostic or antibody laboratory test.
Administration of any COVID-19 vaccine (exclusion)	Administration of the Janssen Ad26.COV2.S vaccine<br>OR<br>Administration of an mRNA vaccine (Pfizer or Moderna):<br>{Any medical claim, pharmacy claim, inpatient hospital encounter, or outpatient hospital encounter with one of the following CPT or NDC codes recorded:<br>CPT codes: 0001A, 0002A, 0012A, 0011A, 91300, 91301<br>NDC codes: 80777-0273-10, 59267-1000-01, 59267-1000-1, 80777-273-10, 59267-1000-2, 59267-1000-02, 59267-1000-03, 80777-0273-15, 80777-0273-98, 80777-273-15, 80777-273-99, 80777-0273-99<br>OR<br>Any retail pharmacy claim for a COVID-19 vaccine with the following manufacturer name:<br>"MOD", "MODERNA", "MODERNA US, INC", "MODERNA US, INC.", "PFIZER", "PFIZER, INC", "PFR" and CVX code is 207 or 208<br><br>Note: CVX codes are codes that indicate the product used in a vaccination and are maintained by the CDC's National Center of Immunization and Respiratory Diseases.

© 2022 Polinski JM et al. *JAMA Network Open.*

eMethods 3. Codes of Pre-exposure Covariates Used for Confounding Adjustment via Propensity Score Matching

Variable name	RWD Definition
Chronic obstructive pulmonary disease (COPD)	Any medical claim, inpatient hospital encounter, or outpatient hospital encounter with one of the following ICD-10-CM codes[1]: I27.8, I27.9, J40, J41, J42, J43, J44, J45, J46, J47, J60, J61, J62, J63, J64, J65, J66, J67, J68.4, J70.1, J70.3
Pulmonary fibrosis	Any medical claim, inpatient hospital encounter, or outpatient hospital encounter with one of the following ICD-10-CM codes[2]: J61, J62.0, J62.8, J63.0, J63.1, J63.2, J63.3, J63.4, J63.5, J63.6, J64, J65, J66.0, J66.1, J66.2, J66.8, J70.1, J84.01, J84.02, J84.03, J84.09, J84.10, J84.111, J84.112, J84.113, J84.114, J84.115, J84.116, J84.117, J84.170, J84.178, J84.2, J84.841, J84.842, J84.843, J84.848, J84.89, J84.9
HIV infection	Any medical claim, inpatient hospital encounter, or outpatient hospital encounter with one of the following ICD-10-CM codes[3]: B20, B97.35, R75, Z21
HIV status (for subgroup definitions)	HIV subgroup definitions based on algorithms defined in[4]: https://www.ahajournals.org/doi/full/10.1161/JAHA.119.013744<br><br>Any medical claim, inpatient hospital encounter, or outpatient hospital encounter occurring from 365 days before cohort entry through cohort entry date with one of the following ICD-10-CM codes[3]: B20, B97.35, R75, Z21<br>OR<br>(2) ≥2 claims for HIV ART medications from 365 days before cohort entry through cohort entry date, defined as claims (medical or pharmacy) or chargemaster encounters with record of administration/prescription for any of the following medications: atazanavir sulfate/cobicistat, didanosine/sodium citrate, efavirenz/lamivudine/tenofovir disoproxil fumarate, elvitegravir/cobicistat/emtricitabine/tenofovir alafenamide, elvitegravir/cobicistat/emtricitabine/tenofovir disoproxil, emtricitabine/rilpivirine hcl/tenofovir alafenamide fumarate, rilpivirine hcl, cobicistat, darunavir eth/cobicistat/emtricitabine/tenofovir alafenamide, dolutegravir sodium/lamivudine, dolutegravir sodium/rilpivirine hcl, tipranavir/vitamin e tpgs, dolutegravir sodium, emtricitabine/rilpivirine hcl/tenofovir disoproxil fumarate, darunavir ethanolate/cobicistat, efavirenz/emtricitabine/tenofovir disoproxil fumarate, elvitegravir, etravirine, tipranavir, abacavir sulfate/dolutegravir sodium/lamivudine, abacavir sulfate/lamivudine/zidovudine, saquinavir mesylate, delavirdine mesylate, saquinavir, didanosine/calcium carbonate/magnesium, enfuvirtide, fosamprenavir calcium, maraviroc, abacavir sulfate/lamivudine, raltegravir potassium, darunavir ethanolate, nelfinavir mesylate, lopinavir/ritonavir, ritonavir, efavirenz, abacavir

© 2022 Polinski JM et al. *JAMA Network Open.*

	sulfate, indinavir sulfate, didanosine, nevirapine, stavudine, zidovudine, lamivudine/zidovudine
Immunocompromised status (as used for subgroups)	Moderately to severely immunocompromised subgroups were defined as those with any diagnosis for active cancer, history of organ/stem cell transplant, primary immunodeficiency (e.g., DiGeorge syndrome, Wiskott-Aldrich syndrome), or HIV infection in the 365d baseline period, AND/OR<br>Recent use (within 60 days of index) of immunosuppressive therapies including high-dose corticosteroids (i.e., ≥20mg prednisone or equivalent per day), transplant-related immunosuppressives, antimetabolites, alkylating agents, and other severely immunosuppressive cancer chemotherapeutics, tumor necrosis factor blockers, and other immunosuppressive biologics. Immunocompromised subgroup definitions based on CDC vaccine guidance for moderately to severely immunocompromised status.[5]<br><br>**Operationalized in claims data as:**<br>Occurrence of Immunocompromised state from blood transplant during 365d baseline period<br>OR<br>Occurrence of Immunocompromised state from organ transplant during 365d baseline period<br>OR<br>Occurrence of HIV infection during 365d baseline period<br>OR<br>Any medical claim, inpatient hospital encounter, or outpatient hospital encounter with one of the following ICD-10-CM codes during 365d baseline period: B20, B97.35, D45, D46.22, D47.1, D47.4, D47.9, D47.Z1, D47.Z9, D61.82, D75.81, D82, D82.0, D82.1, D82.2, D82.3, D82.4, D82.8, D82.9, R75, Z21, Diagnosis for active malignancy among C00-C96 (excluding sub-codes for neoplasms, benign tumors)<br>OR<br>Any medical claim, inpatient hospital encounter, or outpatient hospital encounter with one of the following CPT/HCPCS procedure codes for organ transplantation and related during 365d baseline period: 32852, 32853, 32854, 3490F, 38243, 50365, 50370, A4653, A4671, A4673, A4690, A4700, A4707, A4709, A4712, A4714, A4722, A4725, A4726, A4730, A4737, A4740, A4750, A4755, A4765, A4860, A4890, A4910, A4912, A4913, A4918, E1500, E1510, E1520, E1530, E1540, E1550, E1590, E1592, E1600, E1625, E1634, E1635, S2053, S2054, S2060, S2152, 32851, 33935, 33945, 38240, 38242, 44135, 44136, 47135, 47136, 48554, 50360, A4672, A4674, A4680, A4705, A4706, A4708, A4719, A4720, A4721, A4723, A4724, A4728, A4735, A4736, A4760, A4766, A4802, A4820, A4850, A4870, A4880, A4900, A4901, A4905, A4911, A4914, E1560, E1570, E1575, E1580, E1594, E1610, E1615, E1620, E1630, E1632, E1636, S2065, S2142, S2150

© 2022 Polinski JM et al. *JAMA Network Open.*

	OR<br>Any medical claim, inpatient hospital encounter, or outpatient hospital encounter with one of the following ICD-10-PCS procedure codes for organ transplantation and related during the 365d baseline period:<br>02YA0Z2, 07YM0Z2, 07YP0Z0, 07YP0Z1, 07YP0Z2, 0BYC0Z2, 0BYD0Z1, 0BYD0Z2, 0BYF0Z0, 0BYG0Z1, 0BYG0Z2, 0BYJ0Z0, 0BYJ0Z2, 0BYK0Z2, 0BYL0Z2, 0BYM0Z1, 0BYM0Z2, 0DY50Z0, 0DY50Z1, 0DY50Z2, 0DY60Z2, 0DY80Z0, 0DYE0Z1, 0FY00Z1, 0FY00Z2, 0FYG0Z1, 0FYG0Z2, 0TY00Z0, 0TY00Z2, 0TY10Z1, 0TY10Z2, 30230G1, 30230G2, 30230G4, 30230X3, 30230X4, 30230Y1, 30230Y3, 30233G1, 30233G4, 30233X1, 30233X4, 30233Y1, 30233Y4, 30240AZ, 30240G4, 30240X1, 30240X2, 30240X4, 30240Y1, 30240Y2, 30243AZ, 30243G2, 30243G3, 30243G4, 30243X1, 30243X4, 30243Y2, 30243Y4, 30250G1, 30253G1, 30253X1, 30260G1, 30260X1, 5A1D00Z, 5A1D80Z, 5A1D90Z, BT2900Z, BT290ZZ, BT29Y0Z, BT29YZZ, BT29ZZZ, BT39YZZ, BT39ZZZ, 02YA0Z0, 02YA0Z1, 07YM0Z0, 07YM0Z1, 0BYC0Z0, 0BYC0Z1, 0BYD0Z0, 0BYF0Z1, 0BYF0Z2, 0BYG0Z0, 0BYH0Z0, 0BYH0Z1, 0BYH0Z2, 0BYJ0Z1, 0BYK0Z0, 0BYK0Z1, 0BYL0Z0, 0BYL0Z1, 0BYM0Z0, 0DY60Z0, 0DY60Z1, 0DY80Z1, 0DY80Z2, 0DYE0Z0, 0DYE0Z2, 0FY00Z0, 0FYG0Z0, 0TY00Z1, 0TY10Z0, 30230AZ, 30230G3, 30230X1, 30230X2, 30230Y2, 30230Y4, 30233AZ, 30233G2, 30233G3, 30233X2, 30233X3, 30233Y2, 30233Y3, 30240G1, 30240G2, 30240G3, 30240X3, 30240Y0, 30240Y3, 30240Y4, 30243G1, 30243X2, 30243X3, 30243Y1, 30243Y3, 30250X1, 30250Y1, 30253Y1, 30260Y1, 30263G1, 30263X1, 30263Y1, 3E1M39Z, 5A1D70Z, BT2910Z, BT291ZZ, BT39Y0Z, BT49ZZZ<br>OR<br>Any medical claim, pharmacy claim, inpatient hospital encounter, or outpatient hospital encounter with one of the following NDC Generic Names recorded during the 365d baseline period:<br>azacitidine, bendamustine hcl, busulfan, capecitabine, carmustine, carmustine in polifeprosan 20, chlorambucil, clofarabine, cyclophosphamide, cytarabine, cytarabine liposome/pf, cytarabine/pf, dacarbazine, daunorubicin/cytarabine liposomal, decitabine, decitabine/cedazuridine, diroximel fumarate, floxuridine, fludarabine phosphate, fluorouracil, fluorouracil/adhesive bandage, gemcitabine hcl, gemcitabine hcl in 0.9 % sodium chloride, ifosfamide, ifosfamide/mesna, inebilizumab-cdon, lomustine, melphalan, melphalan hcl, melphalan hcl/betadex sulfobutyl ether sodium, mercaptopurine, nelarabine, ofatumumab, ozanimod hydrochloride, pemetrexed disodium, pralatrexate, satralizumab-mwge, streptozocin, temozolomide, teprotumumab-trbw, thiotepa, upadacitinib, baricitinib, canakinumab/pf, cyclosporine/chondroitin sulfate a sodium, efalizumab, emapalumab-lzsg, guselkumab, infliximab-abda, infliximab-axxq, muromonab-cd3, natalizumab, ocrelizumab, ravulizumab-cwvz, risankizumab-rzaa, sarilumab, siltuximab, tacrolimus/niacinamide, tildrakizumab-asmn,

© 2022 Polinski JM et al. *JAMA Network Open.*

	vedolizumab, anakinra, belatacept, certolizumab pegol, daclizumab, eculizumab, infliximab, infliximab-dyyb, rilonacept, tacrolimus in vehicle base no.238, tacrolimus, micronized, tacrolimus/hyaluronate sodium/niacinamide, ustekinumab, abatacept/maltose, azathioprine sodium, pirfenidone, pomalidomide, secukinumab, teriflunomide, abatacept, alefacept, alemtuzumab, basiliximab, brodalumab, golimumab, mycophenolate mofetil hcl, temsirolimus, tofacitinib citrate, belimumab, siponimod, tacrolimus anhydrous, apremilast, thalidomide, fingolimod hcl, ixekizumab, tocilizumab, dimethyl fumarate, lenalidomide, cladribine, etanercept, adalimumab, methotrexate, mycophenolate sodium, methotrexate/pf, cyclosporine, modified, everolimus, leflunomide, sirolimus, azathioprine, cyclosporine, mycophenolate mofetil, methotrexate sodium/pf, methotrexate sodium, tacrolimus<br>OR<br>Any medical claim, pharmacy claim, inpatient hospital encounter, or outpatient hospital encounter with one of the following CPT/HCPC procedure codes recorded during the 365d baseline period:<br>80158, 80197, C9006, C9020, C9026, C9106, C9110, C9211, C9212, C9230, C9236, C9239, C9249, C9261, C9421, C9455, J0129, J0135, J0202, J0480, J0490, J0638, J1300, J1602, J1628, J2323, J2793, J3245, J3357, J7504, J7507, J7508, J7513, J7515, J7517, J7520, J8530, J9092, J9094, J9095, J9250, J9260, J9310, J9312, J9330, K0120, K0121, K0412, Q2019, Q2044, Q4079, Q5109, S0087, S0162, S0193, S9359, 80169, 80180, 80195, C9126, C9219, C9264, C9286, C9419, C9420, C9436, C9438, J0215, J0485, J0717, J0718, J1438, J1745, J2350, J2860, J3262, J3358, J3380, J7500, J7501, J7502, J7503, J7505, J7511, J7518, J7525, J7527, J8561, J8610, J9010, J9065, J9070, J9080, J9090, J9091, J9093, J9096, J9097, J9311, K0119, K0122, K0123, Q5103, Q5104<br>**Reference and evidence for association:**<br>Immunocompromised subgroup definitions based on CDC vaccine guidance for moderately to severely immunocompromised status.[5]
Immunocompromised state from blood transplant	Any medical claim, inpatient hospital encounter, or outpatient hospital encounter with one of the following ICD-10-CM procedure codes:<br>30230AZ, 30230G1-G4, 30230X1-X4, 30230Y1-Y4, 30233AZ, 30233G1-G4, 30233X1-S4, 30233Y1-Y4, 30240AZ, 30240G1-G4, 30240X1-X4, 30240Y1-Y4, 30243AZ, 30243G1-G4, 30243X1-X4, 30243Y1-Y4, 30250G1, 30250X1, 30250Y1, 30253G1, 30253X1, 30253Y1, 30260G1, 30260X1, 30260Y1, 30263G1, 30263X1, 30263Y1<br>OR<br>ICD-10 diagnosis codes, D84.81, D84.9, D81.1, T86.00, T86.09, Z48.290, Z94.81
Immunocompromised state from organ transplant	Any medical claim, inpatient hospital encounter, or outpatient hospital encounter with one of the following codes[6]:<br>DRG procedure codes: kidney (302), heart (103), lung (495), liver (480)<br>OR

© 2022 Polinski JM et al. *JAMA Network Open.*

	ICD-10 codes: D84.821, D84.9, Z48.21, Z48.22, Z48.23, Z48.24, Z48.280, Z48.288, Z94.0, Z94.1, Z94.2, Z94.3, Z94.4, Z94.82, Z94.83, T86.10, T86.19, T86.20, T86.298, T86.30, T86.39, T86.40, T86.49, T86.818, T86.819, T86.858, T86.859, T86.898, T86.899
Liver disease	Any medical claim, inpatient hospital encounter, or outpatient hospital encounter with one of the following ICD-10-CM codes[1]: B15.9, B16.0, B16.1, B16.2, B16.9, B17.0, B17.10, B17.11, B17.2, B17.8, B17.9, B18, B19.0, B19.10, B19.11, B19.20, B19.21, B19.9, B66.1, B66.3, I85, I86.4, I98.2, K70, K71.0, K71.1, K71.10, K71.11, K71.2, K71.3, K71.4, K71.5, K71.50, K71.51, K71.6, K71.7, K71.8, K71.9, K72, K73, K74, K75.0, K75.1, K75.2, K75.3, K75.4, K75.81, K75.89, K75.9, K76.0, K76.1, K76.2, K76.3, K76.4, K76.5, K76.6, K76.7, K76.8, K76.81, K76.89, K76.9, K77, Q44.6, Z94.4
Malignancies (excluding non-melanoma skin cancer)	Any medical claim, inpatient hospital encounter, or outpatient hospital encounter with one of the following ICD-10-CM codes[1]: C00, C01, C02, C03, C04, C05, C06, C07, C08, C09, C10, C11, C12, C13, C14, C15, C16, C17, C18, C19, C20, C21, C22, C23, C24, C25, C26, C30, C31, C32, C33, C34, C37, C38, C39, C40, C41, C43, C45, C46, C47, C48, C49, C50, C51, C52, C53, C54, C55, C56, C57, C58, C60, C61, C62, C63, C64, C65, C66, C67, C68, C69, C70, C71, C72, C73, C74, C75, C76, C81, C82, C83, C84, C85, C88, C90, C91, C92, C93, C94, C95, C96, C97
Moderate-to-severe asthma	Any medical claim, inpatient hospital encounter, or outpatient hospital encounter with one of the following ICD-10-CM codes[7]: J45.40, J45.41, J45.42, J45.50, J45.51, J45.52
Cerebrovascular disease	Any medical claim, inpatient hospital encounter, or outpatient hospital encounter with one of the following ICD-10-CM codes[1]: G45, G46, H34.0, I60, I61, I62, I63, I64, I65, I66, I67, I68, I69
Chronic kidney disease	Any medical claim, inpatient hospital encounter, or outpatient hospital encounter with one of the following ICD-10-CM codes[1]: I12.0, 13.1, N03.2, N03.3, N03.4, N03.5, N03.6, N03.7, N05.2, N05.3, N05.4, N05.5, N05.6, N05.7, N18, N19, N25.0, Z49.0, Z49.1, Z49.2, Z94.0, Z99.2
Hypertension	Any medical claim, inpatient hospital encounter, or outpatient hospital encounter with one of the following ICD-10-CM codes[1]: I10, I11, I12, I13, I15, I16, I27.0, I27.1, I27.20, I27.21, I27.22, I27.23, I27.24, I27.29, I67.4, I87.301, I87.302, I87.303, I87.309, I87.311, I87.312, I87.313, I87.319, I87.31, I87.321, I87.322, I87.323, I87.329, I87.331, I87.332, I87.333, I87.339, I87.391, I87.392, I87.393, I87.399, I97.3, K76.6, R03.0, G93.2
Serious heart conditions	Any medical claim, inpatient hospital encounter, or outpatient hospital encounter with one of the following ICD-10-CM codes[1,8]: 109.9, 111,

© 2022 Polinski JM et al. *JAMA Network Open.*

	113, 113.2, I25.5, I42.0, I42.5-I42.9, I43, I50, P29.0, I20, I21, I22, I23, I24, I25, I05, I06, I07, I08, I20.0, I20.1, I20.8, I20.9, I23.7, I24.0, I24.1, I24.8, I24.9, I25.10, I25.110, I25.111, I25.118, I25.119, I25.2, I25.5, I26.01, I26.02, I26.09, I26.90, I26.92, I26.99, I27.0, I27.1, I27.2, I27.81, I27.82, 414.1, 414.11, 414.12, 414.19, 429.1, 429.2, 429.3, 429.5, 429.6, 429.71, 429.79, 429.81
Obesity	Any medical claim, inpatient hospital encounter, or outpatient hospital encounter with one of the following ICD-10-CM codes[1,9]: Z68.30, Z68.31, Z68.32, Z68.33, Z68.34, Z68.35, Z68.36, Z68.37, Z68.38, Z68.39, Z68.41, Z68.42, Z68.43, Z68.44, Z68.45, Z68.51, Z68.52, Z68.53, Z68.54, Z98.0, Z98.84, E66
Sickle Cell Disease	Any medical claim, inpatient hospital encounter, or outpatient hospital encounter with one of the following ICD-10-CM codes: D57
Thalassemia	Any medical claim, inpatient hospital encounter, or outpatient hospital encounter with one of the following ICD-10-CM codes: D56
Type 1 diabetes mellitus	Any medical claim, inpatient hospital encounter, or outpatient hospital encounter with one of the following ICD-10-CM codes[1]: E10
Type 2 diabetes mellitus	Any medical claim, inpatient hospital encounter, or outpatient hospital encounter with one of the following ICD-10-CM codes[1]: E11
Type 2 diabetes (for subgroup definitions)	Any medical claim, inpatient hospital encounter, or outpatient hospital encounter occurring from 365 days before cohort entry through cohort entry date with one of the following ICD-10-CM codes[1]: E11
Neurologic conditions	Any medical claim, inpatient hospital encounter, or outpatient hospital encounter with one of the following ICD-10-CM codes[3,10]: F01, F00, G00.0, G00.1, G00.2, G00.3, G00.8, G00.9, G01-G14, G20-G26, G30-G32, G31 (exclude G31.2), G35-37, G40, G43-G47, G50-G65, G70-G73, G80-G83, G89-99, G45.8, G45.9, I60-I63, I65-I69, F03, F04, R29, R41, S06, Z86.73, Z87.820, F02.80, F02.81, F05, F06.0, F06.8, F10.27, F19.97
Number of pharmacy claims	The count of events occurring in the “Pharmacy Claims” table over the baseline period
Number of medical claims	The count of events occurring in the “Medical Claims” table over the baseline period

© 2022 Polinski JM et al. *JAMA Network Open.*

Recent medical claims	True if any event begins in the “Medical Claims” table in the 60 days prior to index
Recent pharmacy claim	True if any event begins in the “Pharmacy Claims” table in the 60 days prior to index
Age	Age defined as continuous numeric variable for propensity score modeling.
Sex	Male/Female
Comorbidity score (exact)	Gagne Combined Comorbidity Index
Calendar month of index date	The calendar month in which the index date occurs may be one of the following: 2021-03, 2021-04, 2021-05, 2021-06, 2021-07
State	Classified based on U.S. state, D.C., or miscellaneous U.S. territory recorded on index date.

© 2022 Polinski JM et al. *JAMA Network Open.*

eMethods 4. Vaccine Underreporting and Correction

Assumptions for vaccine under-recording corrections were based on comparison of vaccination rates in HealthVerity to those observed in CDC national data and the linked Louisiana State Vaccine Registry.

Vaccine under-recording: comparison to national CDC data

The CDC reported nationwide 168.8 million fully vaccinated people out of 283.7 million people 12 years and older in the US (~59.5%) as of August 17, 2021, the last day for which individuals were eligible for cohort entry in our study. Within the HealthVerity dataset used for the current study, we observed 60.4 million individuals with any COVID-19 vaccination out of 168.8 million people included in the dataset (~35.7%). The under-recording of COVID-19 vaccinations in the national claims data used for this study can thus be approximated by 1-35.7%/59.5% ≈ 40% under-recording, which equates to 60% sensitivity of exposure assessment.

Vaccine under-recording: comparison to Louisiana State Vaccine Registry

Through patient-level linkage of Louisiana State Vaccine Registry information to the HealthVerity dataset, we can more precisely quantify the level of under-recording for a subset of the national claims dataset. Among the Louisiana population without vaccination events in HealthVerity (presumed unvaccinated), 43% appeared as vaccinated within the linked Louisiana State Registry database. Assuming the Louisiana State Vaccine Registry represents a complete recording of vaccination status of individuals linked across datasets, this yields a sensitivity of exposure assessment of ~57% within the Louisiana HealthVerity population or an under-recording of ~43%. The derived under-recording estimates were similar across age 65+/- (both ~43%), immunocompromised (~44%), and HIV positive (~52%) subgroups.

Correction for vaccine under-recording

Such reduced sensitivity of exposure assessment leads to an underestimation of the true effectiveness, as our unvaccinated comparator population likely includes patients who were vaccinated at mass vaccination sites, pharmacies, and other organizations without submitting claims to insurance companies and thus our study data source.[11] Uncorrected VE estimates shown in main manuscript tables are based on an implicit assumption of complete capture (no under-recording) of vaccine events, and thus represent a lowest bound of VE for each analysis. For primary analysis bias-corrected effect estimates, we conservatively assumed an under-reporting of 40% (sensitivity of 60%).

Assuming a 40% estimated proportion of vaccination under-reporting in claims data, we used standard algebraic methods to correct relative risk estimates assuming 100% specificity of vaccination recording.[12] Observed and corrected relative risks and 95% confidence intervals were calculated by specifying a 61-day fixed follow-up period that began 14 days post-index among vaccinated and 1:1 PS matched unvaccinated patients. From this fixed follow-up analysis, specific bias correction factors were derived for each outcome and all subgroups of interest, and then applied to hazard ratio and rate ratio-based effectiveness estimates from the primary analysis.[11]

© 2022 Polinski JM et al. *JAMA Network Open.*

Rather than deriving separate under-reporting rates for each subgroup and analysis within the study, we opted to apply one consistent adjustment factor (assuming 40% under-recording) to all corrected estimates, with sensitivity analyses examining the effect of varying this assumption. **Supplemental Tables S4** and **S5** show corrected VE estimates across a range of assumed under-recording scenarios, from complete capture (no under-recording) to 70% under-recording. We hope to refine this correction approach for future monthly refreshes through further linkage to state registry information and consideration of changes in under-reporting rates by time and geography.

© 2022 Polinski JM et al. *JAMA Network Open.*

eMethods 5. Month-by-Month Effectiveness Analyses

To assess vaccine effectiveness over calendar time, we examined incident rate ratios separately for each calendar month within the overall National Cohort and High-Delta-incidence States (**Fig 3**). **Table 2** also shows effectiveness estimates for the combined period June to August 2021 for high-Delta-incidence states, given the emergence of the Delta variant during this period.

Only outcome events and eligible person-time occurring within each person's follow-up period for main effectiveness analyses (March 2021 to August 2021) were included in month-level incident rate ratio calculations. Persons could contribute eligible person-time for multiple months, and/or for partial months, but were excluded from monthly person-time totals if censored from analysis. **Figure S3** shows how month-by-month person-time denominators were derived for 2 hypothetical study participants.

Using this approach, monthly incident rates were calculated as the (number of outcome events beginning in each calendar month) / (the total number of person-years within each month for which persons were eligible for outcomes). Incident rate ratios were calculated for each month as the ratio of outcome rates in exposed vs unexposed patients, and effectiveness estimates were then corrected for vaccine under-recording in claims data using the methods described in **Suppl. S4**.

© 2022 Polinski JM et al. *JAMA Network Open.*

eMethods 6. HealthVerity Data Linkage Overview

Note: HealthVerity data linkage information is from HealthVerity proprietary white paper

HealthVerity De-Identification Overview:

HealthVerity uses a mixture of techniques to achieve accurate record linkage, including both straight hashing and Bloom filters (probabilistic). This allows us to be robust to errors and variations in text fields while maintaining a high level of patient privacy.

For fields that are purely numeric, such as date-of-birth or Social Security number (SSN), the data is subjected to a straight hash. We use SHA-256 hashing with a salt provided by a 3rd party server (not owned or managed by HealthVerity).

Free text fields, such as the patient's name, are prone to small errors and variations that make straight hashing a very brittle approach. We use a specific form of Bloom filters based on Durham et al.[13] to encode these fields into a special hash that supports probabilistic matching in the presence of small variations. We extend the existing work as described below to provide additional safeguards on the patient's privacy.

Bloom Filters:

Achieving accurate record linkage using such hashes is non-trivial because a patient's record often contains typographical and semantic errors.[14] To overcome this problem, for fields that are likely to contain typographical errors and are not directly attackable using a constraint satisfaction program. HealthVerity will split a patient's identifier into a series of *n*-grams (an example of which is shown in Figure 1), whereby the patient's identifiers are split into a series of overlapping substrings that are subject to a set of hash functions and mapped into a Bloom Filter (BF), which is a binary array of a fixed length.

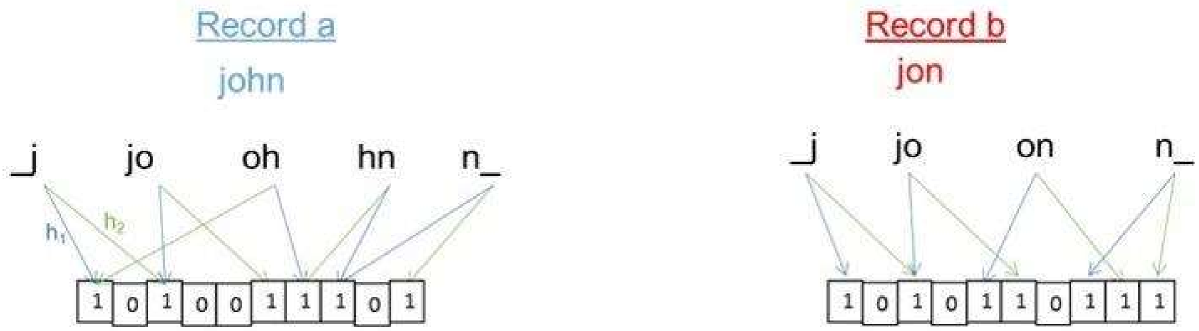

eMethods 6 Figure 1. Bloom filters for two names (John and Jon) with two hash functions.

When each field (e.g., forename, surname, etc.) is mapped into such a structure, it is referred to as a field-level Bloom filter (FBF). It has been shown that FBFs over common patient identifiers can be highly accurate.[15] Yet, it has also been shown that FBF encodings can be insecure because they reveal frequency distributions for field values (e.g., specific names) and bit positions (i.e., *n*-

© 2022 Polinski JM et al. *JAMA Network Open.*

grams). Specifically, it has been shown that parameter settings recommended in Schnell et al.,[15] which consists of 2-grams, a filter length of 500 bits, and 15 hash functions, can be cracked to leak names and geocodes (e.g., 5-digit ZIP) when the adversary has access to some global frequency distribution of such information [Kuzu 2011], such as a voter registration list or address directory.[16] It should be note that evidence suggests the identifiers available to an attacker may not be of the same frequency distribution as those observed in the FBFs.[16] However, it was shown that, even though the distributions may not be equivalent, an attacker can allow for weaker constraints in their cryptanalysis and commit a successful cracking of the system. Given that it is not always possible to determine what data sources an adversary may have access to, this risk analysis aims to account for the worst-case scenario (i.e., when the hashed records and information available to the adversary are derived from the same resource), and thus ensure a system is robust against a constraint satisfaction- based cryptanalysis.

At the same time, it was further shown that as *n* grows, the likelihood of a successful attack diminishes. This is because as *n* increases, so too does the number of distinct n-grams, which leads to a smaller quantity of frequency-related information to commit a successful cryptanalysis. While this observation suggests a simple strategy to improve security, it was also found that *n* and linkage accuracy were inversely correlated. Thus, a more principled mechanism, independent of *n* has been developed to increase the security of BF-based encodings.

Specifically, HealthVerity uses a record-level Bloom filter (RBF), which merges multiple FBFs to prevent cryptanalysis.[13] HealthVerity has implemented this by mapping all the FBFs into the same bit space, such that it is difficult to discern from which fields such bits were derived. However, each FBF is still computed separately, using a distinct set of salts.

Implementation:

The above section explains the general RBF data structure for the pseudonyms generated for use by HealthVerity. However, it does not provide details on how the RBF is generated or what specifically it is composed of. To do so, let us consider the process of data generation.

Figure 2 provides an illustration for how hashing and data transmission process works. It should be noted that the salt for the system is generated by a 3rd party server (not owned or managed by HealthVerity). This is critical because it ensures that HealthVerity cannot run a dictionary attack against the system.

© 2022 Polinski JM et al. *JAMA Network Open.*

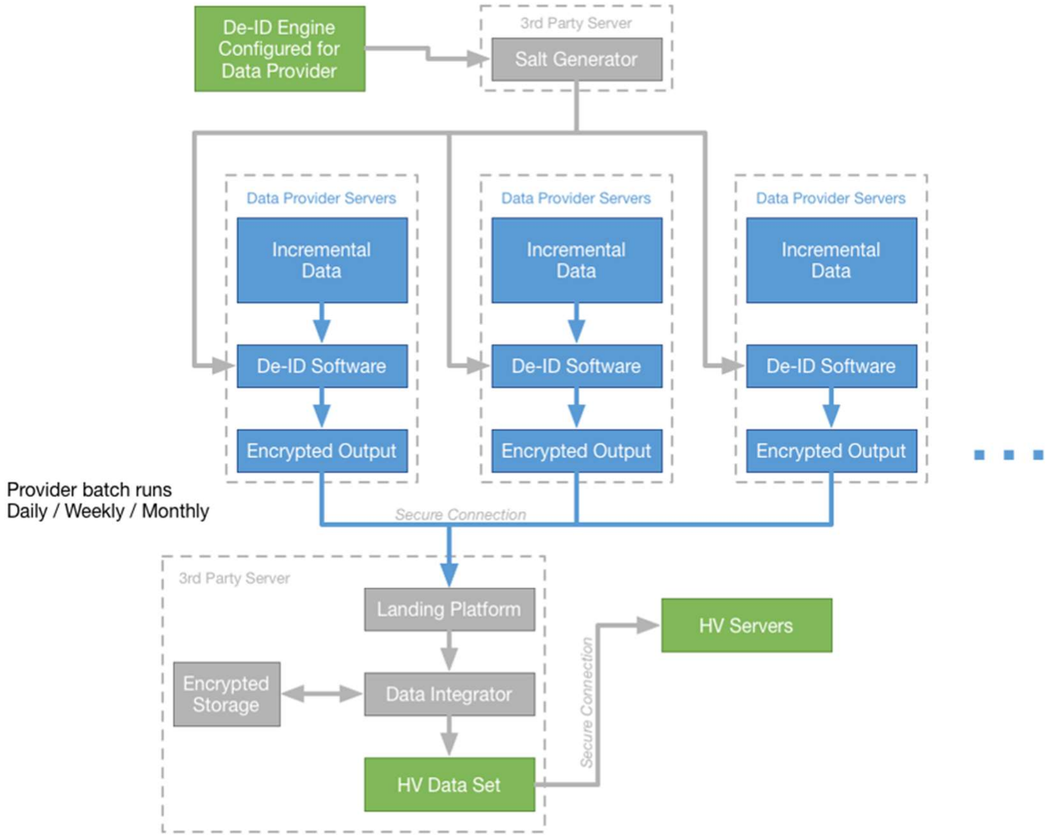

eMethods 6 Figure 2. General architecture for RBF generation.

Additionally, note that the tokens from the de-identification (DeID) are encrypted and sent to a different 3rd party to perform the record matching process. These are encrypted at rest, and there is no PII data held on this 3rd party server. At no time does HealthVerity have access to any of the DeID tokens, and the only output that we receive for each record is a single HealthVerity ID number, based on the result of the matching.

The RBF is a composite over multiple fields, corresponding to the first and last (i.e., forename and surname) of the patient. The parameters for the RBF is set according to the strategy laid out in Durham et al.,[13] specifically:

1. All names are truncated to ensure that the total length of the first and last name does not exceed 20 characters. Preference is given truncating the first name initially unless the last name exceeds 19 characters by itself. In that case, the first initial of the first name is used, along with the first 19 characters of the last name.
2. All names are translated into a set of lowercase trigrams.
3. Names are padded with two null characters at the beginning and end, providing a total of N+2 trigrams for a name with N characters.

4. Each trigram is subject to 10 rounds of hashing, using the salts provided by a 3rd party.
    1. The first and last names use a different set of salts. The salts remain consistent for all trigrams in the first name, and for all trigrams in the last name.
    2. The first name and last name are mapped into the same bit array.

c. The first round of hashing for a trigram is on the trigram plus the salt. The nine subsequent rounds of hashing are on the output of the previous round plus the next salt.

5. The RBF consists of 208 bits. This length is chosen to ensure that (on average) 50% of the bits are set to 1, derived from a representative sample of data.
6. To further protect the name length, the RBF will have all the bits inverted if the total number of bits set to 1 falls below 104 (50%).

Probabilistic Matching:

The RBF method described above allows HealthVerity to implement a highly reliable method of anonymous record linkage. This is because two names that differ by a single typographic error will have RBF hashes that still share most of the same bits. The number of differing bits forms a narrow probability distribution function, allowing us to accurately assess the likelihood that two RBF fields correspond to the same patient.

Using approximate field matching with the RBFs, in concert with additional Bayesian methods to address missing data and changing field values (e.g., a patient moves to a new zip code), These patterns are learned from the observed data and constantly updated within the matching engine. All the probabilities are normalized by the observed frequency of the underlying tokens, so that matching the tokens for a common name like John Smith carries less evidentiary weight than matching the tokens for a truly unique name. The probabilities for the individual tokens are combined into a full joint conditional probability for each patient, and the results normalized across the entire population. HealthVerity currently has a database of tokens for 330M individuals in the United States that new queries are matched against, including numerous variations of the individual tokens. HealthVerity significantly improves the matching accuracy over state-of-the-art heuristic matching methods.

Performance and Validation:

The use of Bloom filters produces a significant improvement in matching accuracy, as traditional means of hashing are fully deterministic and as a result are susceptible to significant false negative errors in matching due to source data variance. Based on both simulation and real-world use cases with current client data, traditional methods of deterministic matching can produce a false negative rate of between 7-35%.

The hashing components have undergone extensive analysis for frequency patterns and known attacks as a part of the expert determination in our HIPAA expert determination certifications performed by multiple separate parties (Malin, 2016/2018, El Emam, 2017). In addition, Bloom filter hashing is well established in the literature.

HealthVerity uses probabilistic matching, with probabilities normalized across the population. Currently, there is support for reporting the top ten match candidates for an individual, along with their relative probabilities. The default configuration is set to a threshold to prefer false negatives over false positives by a 10:1 ratio. This threshold may be set differently at a system level, or dynamically changed by the individual users through filtering of the top candidates.

HealthVerity has performed a series of extensive tests of the sensitivity and specificity of our system in simulation, as well as side-channel analysis of the real-world matching rates. Simulation tests were performed on the public data sets of 11M+ real-world individuals, under a variety of conditions and assumptions. Naturally, the results of these tests depend heavily on conditions such as data fidelity, error rates, demographic skews and shifts, and available fields. Under data conditions typical of medical records, the system consistently performed at 98.0% sensitivity and 99.8% specificity, regardless of demographics and scale.

Validating the results on real-world data in process is much more difficult, particularly in a privacy preserving setting. Fortunately, there are side-channels for analysis that allow us to indirectly identify sensitivity and specificity. Comparing identities to census data, in aggregate and in crosstabs, can identify overcounts due to identity fragmentation. Move rates between geographic regions can identify false negatives from a particularly challenging situation for matching. Even in regions where HealthVerity may not have full coverage of a population, the amount of variance in predicted demographic ratios versus ratios observed in the HVIDs can be used to bound the sensitivity rates. A variety of these methods generally agree on an actual sensitivity of 98.2%. Conversely, specificity can be estimated using conflict rates, such as when a single identity has mutually exclusive procedures or diagnoses, the most intuitive of which is post-mortem activity. HealthVerity has access to month of death indicators for a significant portion of the HVIDs. While some medical claims may still be filled in the months immediately following death, any activity more than six months later is an indicator that multiple individuals were given the same HVID. Multiple independent estimates using these types of mutual exclusivity consistently provide an estimated specificity of 99.6-99.7%. One empirical study with limited data was performed with a data partner, using hashed 4-digit SSNs. Real data on approximately 8,000 individuals with entries from multiple data sources were run through HealthVerity's system, and the results were compared using the hashed SSNs as a surrogate for identity. After accounting for possible coincidental matches (since there were less than 10,000 possible hashed SSNs), the sensitivity was estimated at 98.5% and the specificity was estimated at 99.7%. While the sample size was small, these numbers continue to agree with the independent estimates we get from simulated and from side-channel analyses.

© 2022 Polinski JM et al. *JAMA Network Open.*

eMethods 7. Representativeness of Study Population as Compared With US Census Bureau Estimates

	Study population identified in HealthVerity dataset		
	Ad26.COV2.S-vaccinated	Matched unvaccinated	US Census Bureau estimates (2020)
**N**	**422,034**	**1,645,397**	**331,501,080**
**Insurance coverage**			
Commercial	236,759 (56.1%)	910,734 (55.4%)	66.5%
Medicaid	58,700 (13.9%)	248,222 (15.1%)	17.8%
Medicare	106,924 (25.3%)	437,478 (26.6%)	18.4%
**Region**			
Northeast	56,655 (13.4%)	220,319 (13.4%)	17.4%
Midwest	95,301 (22.6%)	370,693 (22.5%)	20.8%
South	177,041 (41.9%)	692,261 (42.1%)	38.1%
West	93,037 (22.0%)	362,124 (22.0%)	23.7%

Footnote: Insurance coverage in the HealthVerity study population is based on evidence of any coverage from 365 days pre-index to index date, inclusive. Not mutually exclusive; individuals can have evidence of >1 insurance type. Sums to less than 100% due to uninsured and unknown insurance statuses.

U.S. Census Bureau information sourced from the Annual Social and Economic Supplement (ASEC) of the 2020-2021 Current Population Survey (CPS).[17]

© 2022 Polinski JM et al. *JAMA Network Open.*

eReferences.

1. Quan H, Sundararajan V, Halfon P, et al. Coding Algorithms for Defining Comorbidities in ICD-9-CM and ICD-10 Administrative Data: Medical Care 2005;43(11):1130–9.

2. Jones N, Schneider G, Kachroo S, Rotella P, Avetisyan R, Reynolds MW. A systematic review of validated methods for identifying pulmonary fibrosis and interstitial lung disease using administrative and claims data: DETECTION OF PULMONARY FIBROSIS IN CLAIMS. Pharmacoepidemiol Drug Saf 2012;21:256–60.

3. Fireman B, Toh S, Butler MG, et al. A protocol for active surveillance of acute myocardial infarction in association with the use of a new antidiabetic pharmaceutical agent: ACTIVE SURVEILLANCE OF ACUTE MYOCARDIAL INFARCTION. Pharmacoepidemiol Drug Saf 2012;21:282–90.

4. Rosenson RS, Hubbard D, Monda KL, et al. Excess Risk for Atherosclerotic Cardiovascular Outcomes Among US Adults With HIV in the Current Era. Journal of the American Heart Association 2020;9(1):e013744.

5. Interim Clinical Considerations for Use of COVID-19 Vaccines | CDC [Internet]. 2021 [cited 2021 Oct 4];Available from: https://www.cdc.gov/vaccines/covid-19/clinical-considerations/covid-19-vaccines-us.html

6. Beckman KD. Coding Common Respiratory Problems in ICD-10. FPM 2014;21(6):17–22.

7. Quinlan SC, Morton LM, Pfeiffer RM, et al. Increased Risk for Lymphoid and Myeloid Neoplasms in Elderly Solid-Organ Transplant Recipients. Cancer Epidemiol Biomarkers Prev 2010;19(5):1229–37.

8. Lindgren M, Robertson J, Adiels M, et al. Elevated resting heart rate in adolescent men and risk of heart failure and cardiomyopathy. ESC Heart Failure 2020;7(3):1178–85.

9. Caplan EO, Kamble PS, Harvey RA, et al. Positive predictive value between medical-chart body-mass-index category and obesity versus codes in a claims-data warehouse. Current Medical Research and Opinion 2018;34(1):117–21.

10. Fujiyoshi A, Jacobs DR, Alonso A, Luchsinger JA, Rapp SR, Duprez DA. Validity of Death Certificate and Hospital Discharge ICD Codes for Dementia Diagnosis: The Multi-Ethnic Study of Atherosclerosis. Alzheimer Disease & Associated Disorders 2017;31(2):168–72.

11. Rothman KJ, Lash TL, VanderWeele TJ, Haneuse S. Modern epidemiology. Fourth edition. Philadelphia: Wolters Kluwer; 2021.

12. Lash TL, Fox MP, Fink AK. Applying Quantitative Bias Analysis to Epidemiologic Data. 1st ed. New York: Springer; 2009.

13. Durham EA, Kantarcioglu M, Xue Y, Toth C, Kuzu M, Malin B. Composite Bloom Filters for Secure Record Linkage. IEEE Trans Knowl Data Eng 2014;26(12):2956–68.

14. Hernandez MAH, Stolfo SJ. Real-world Data is Dirty: Data Cleansing and The Merge/Purge Problem. Data mining and knowledge discovery 1998;2(1):9-37.

© 2022 Polinski JM et al. *JAMA Network Open.*

15. Schnell R, Bachteler T, Reiher J. Privacy-preserving record linkage using Bloom filters. BMC Med Inform Decis Mak 2009;9(1):41.

16. Kuzu M, Kantarcioglu M, Durham E, Malin B. A Constraint Satisfaction Cryptanalysis of Bloom Filters in Private Record Linkage. 2011.

17. U.S. Census Bureau (2021). Health Insurance Coverage in the United States: 2020. Annual Social and Economic Supplement (ASEC) of the 2021 Current Population Survey (CPS). Retrieved from: https://www.census.gov/library/publications/2021/demo/p60-274.html

© 2022 Polinski JM et al. *JAMA Network Open.*

eFigure 1. Study Design Diagram

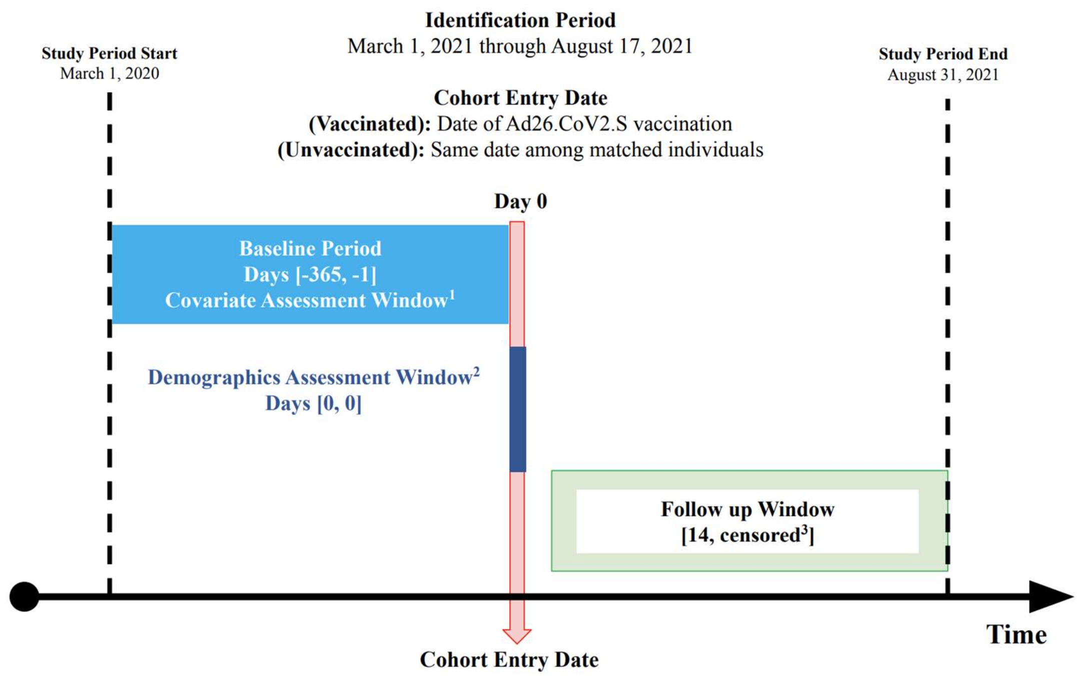

1 - Number of pharmacy/medical claims and comorbidities
2 - Age, sex and state
3 - Day 14 through observed endpoint or censoring.
Individuals with censoring events between cohort entry date and start of follow-up on day 14 were excluded from follow-up analyses.

© 2022 Polinski JM et al. *JAMA Network Open.*

eFigure 2. Schoenfeld Residuals Plot for Primary Analysis in National Cohort and High Delta-Incidence States

S2a) Plot of Schoenfeld residuals for any recorded COVID-19 infection in the National Cohort

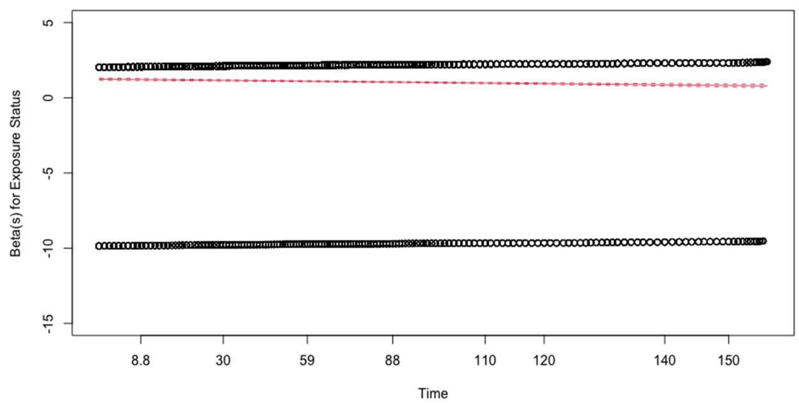

S2b) Plot of Schoenfeld residuals for COVID-19-related hospitalization in the National Cohort

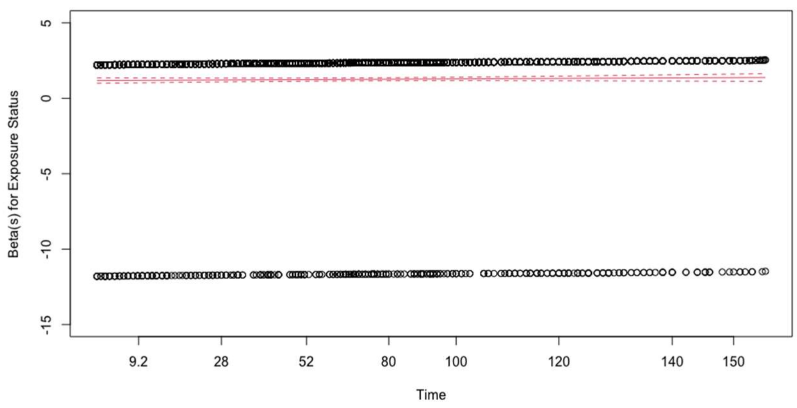

S2c) Plot of Schoenfeld residuals for any recorded COVID-19 infection among High-Delta-incidence States

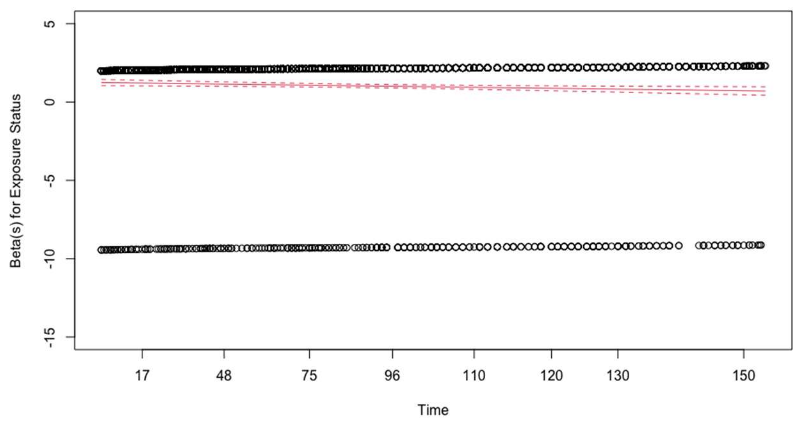

S2d) Plot of Schoenfeld residuals for COVID-19-related hospitalization among High-Delta-incidence States

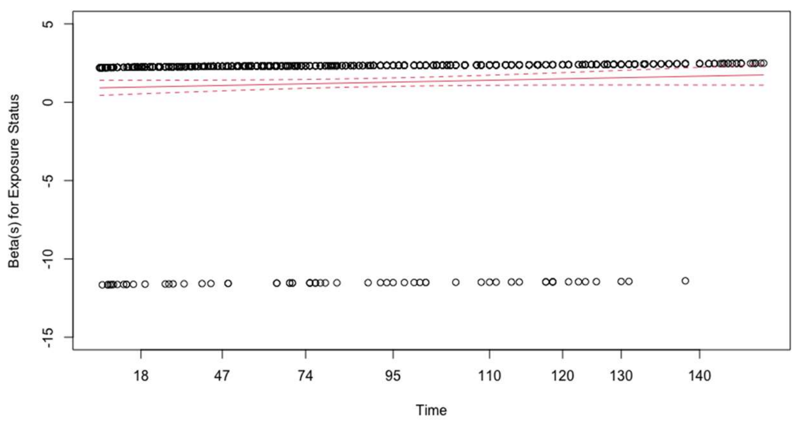

© 2022 Polinski JM et al. *JAMA Network Open.*

eFigure 3. Diagrams for Methods Used to Calculate Month-Specific Effectiveness Estimates

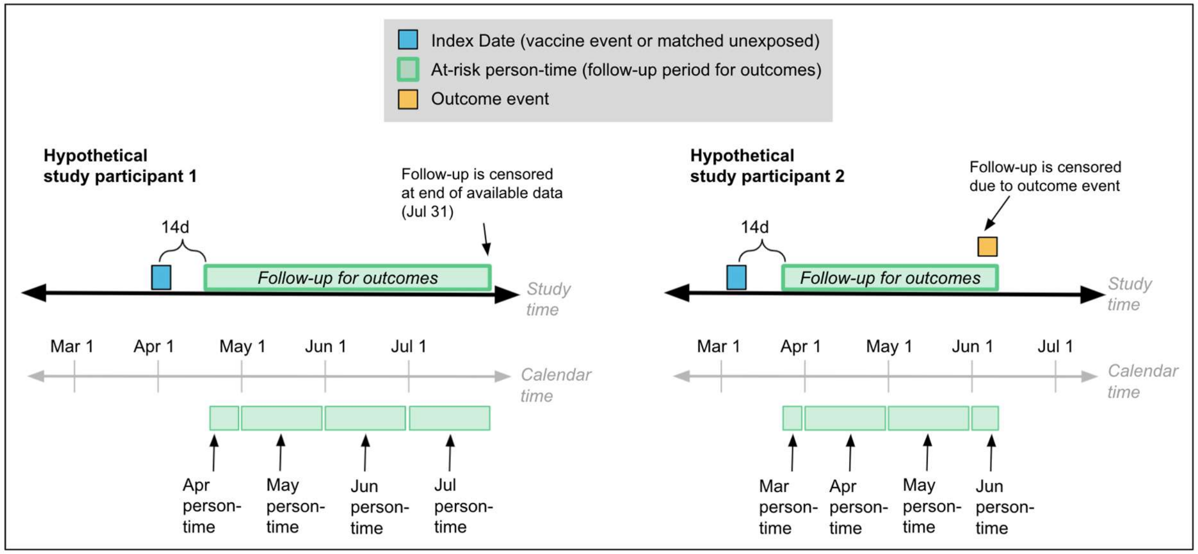

Hypothetical study participant 1 *contributes to separate person-time denominators for part of April, May, June, and July. Person 1 has no outcome events during this eligible time window.*

Hypothetical study participant 2 *contributes to separate person-time denominators for part of March, April, May, and part of June, and an outcome event in June.*

© 2022 Polinski JM et al. *JAMA Network Open.*

eFigure 4. COVID-19–Related Outcomes by Time Since Vaccination (+14 Days) for Subgroups Within National Cohort (Kaplan-Meier Plots)

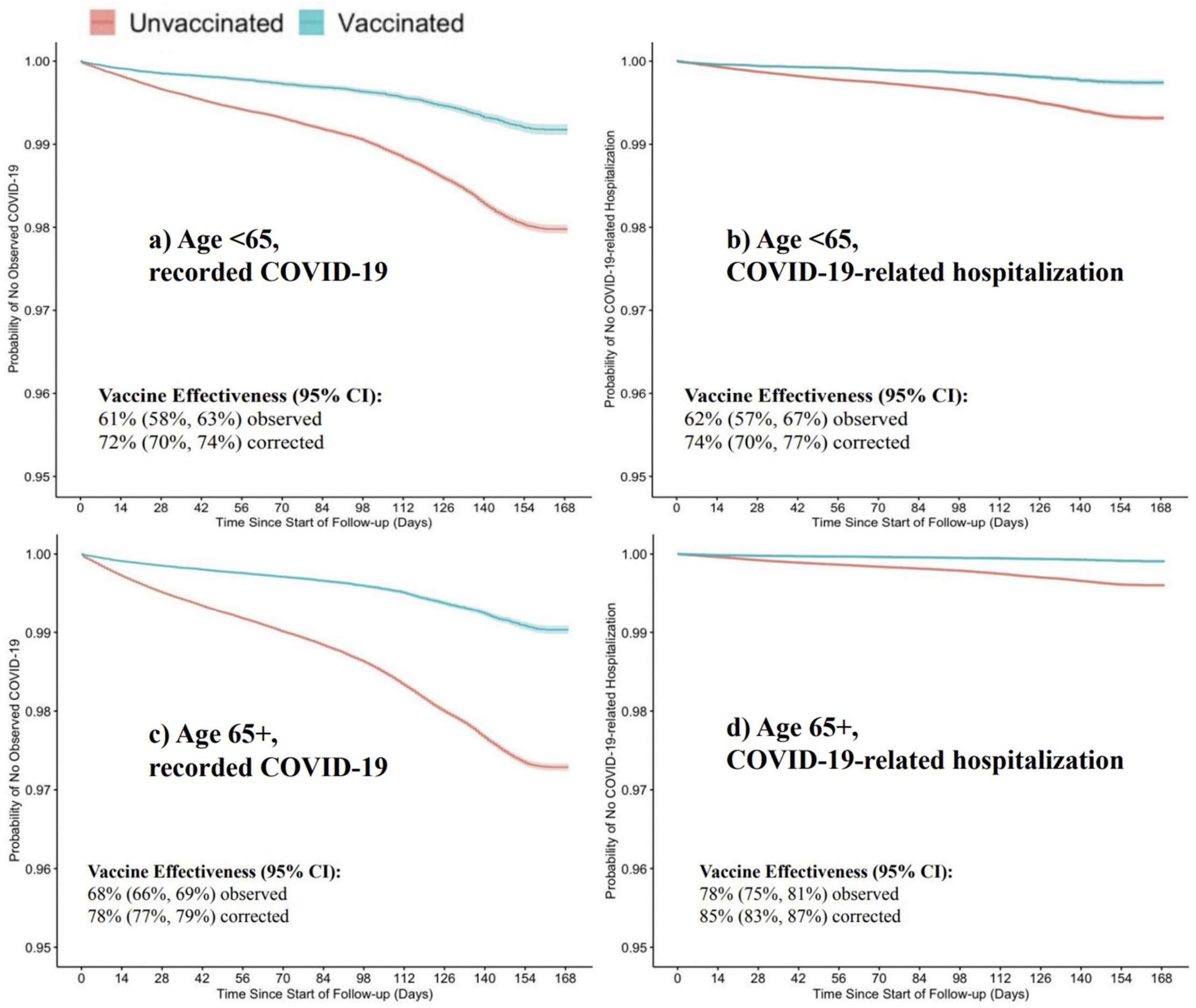

© 2022 Polinski JM et al. *JAMA Network Open.*

eFigure 4, continued.

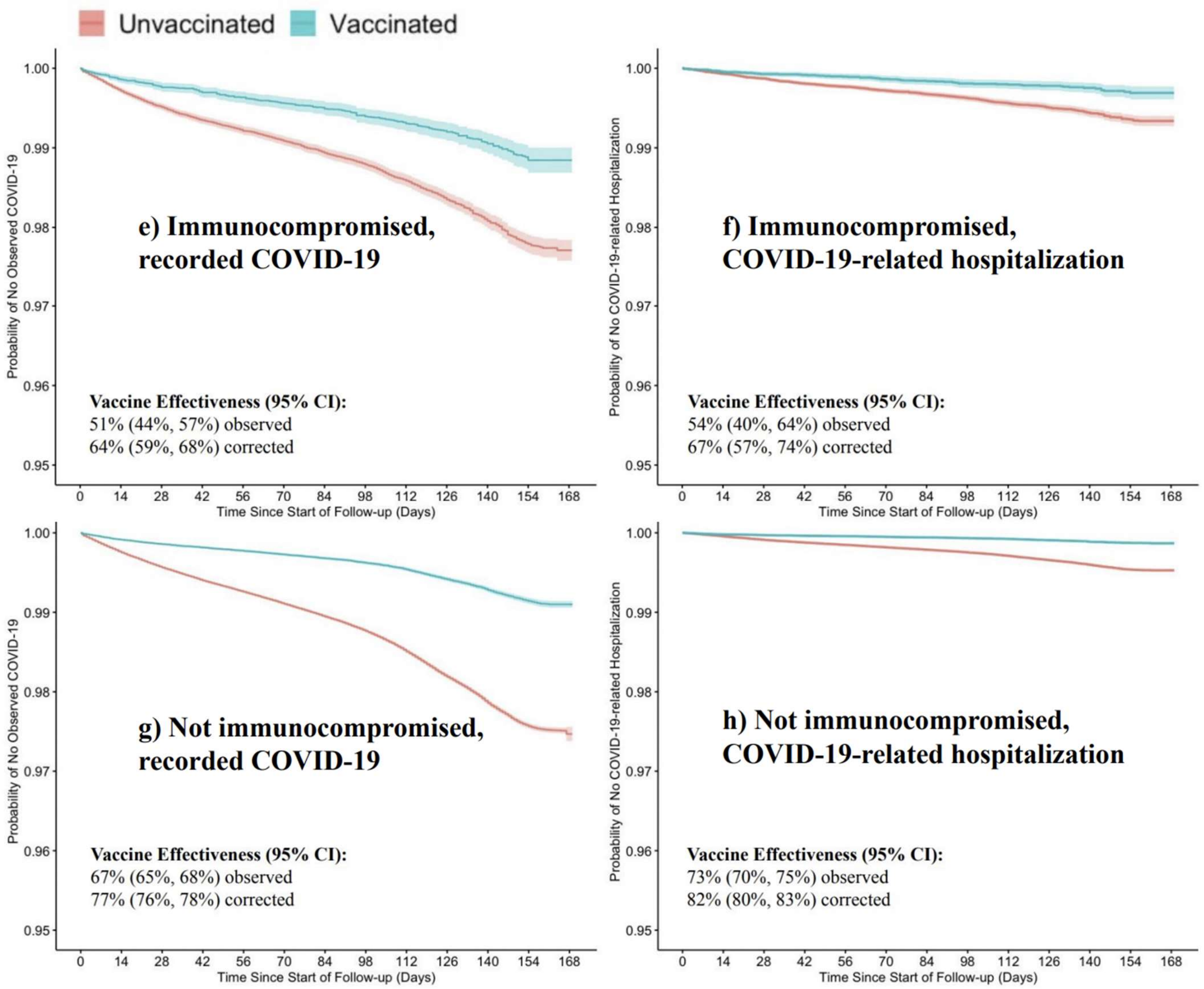

© 2022 Polinski JM et al. *JAMA Network Open.*

eFigure 4, continued.

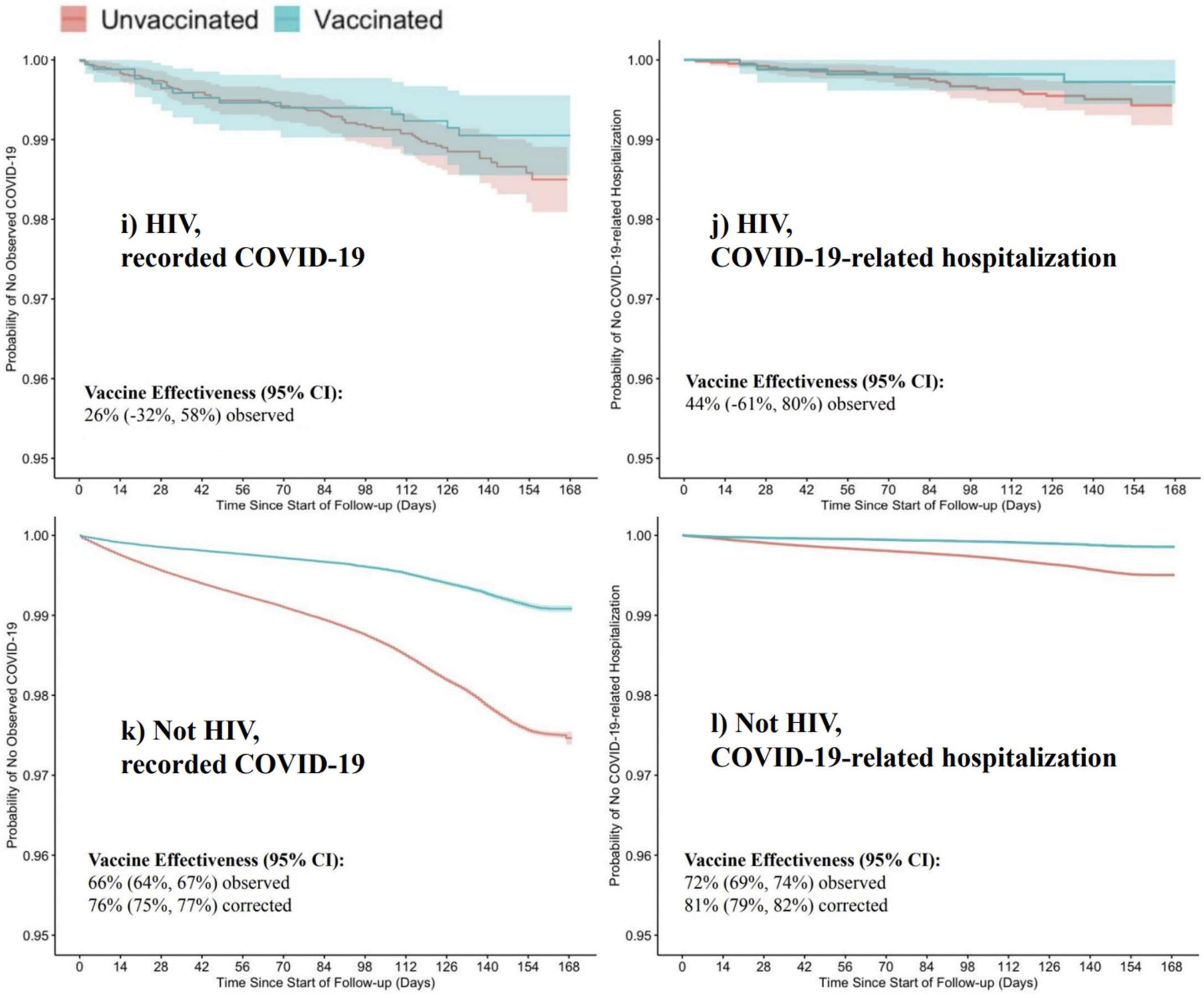

Insufficient outcome counts for application of under-correction methods within the HIV positive subgroup (panels **S2i** and **S2j**).

© 2022 Polinski JM et al. *JAMA Network Open.*

eFigure 4, continued.

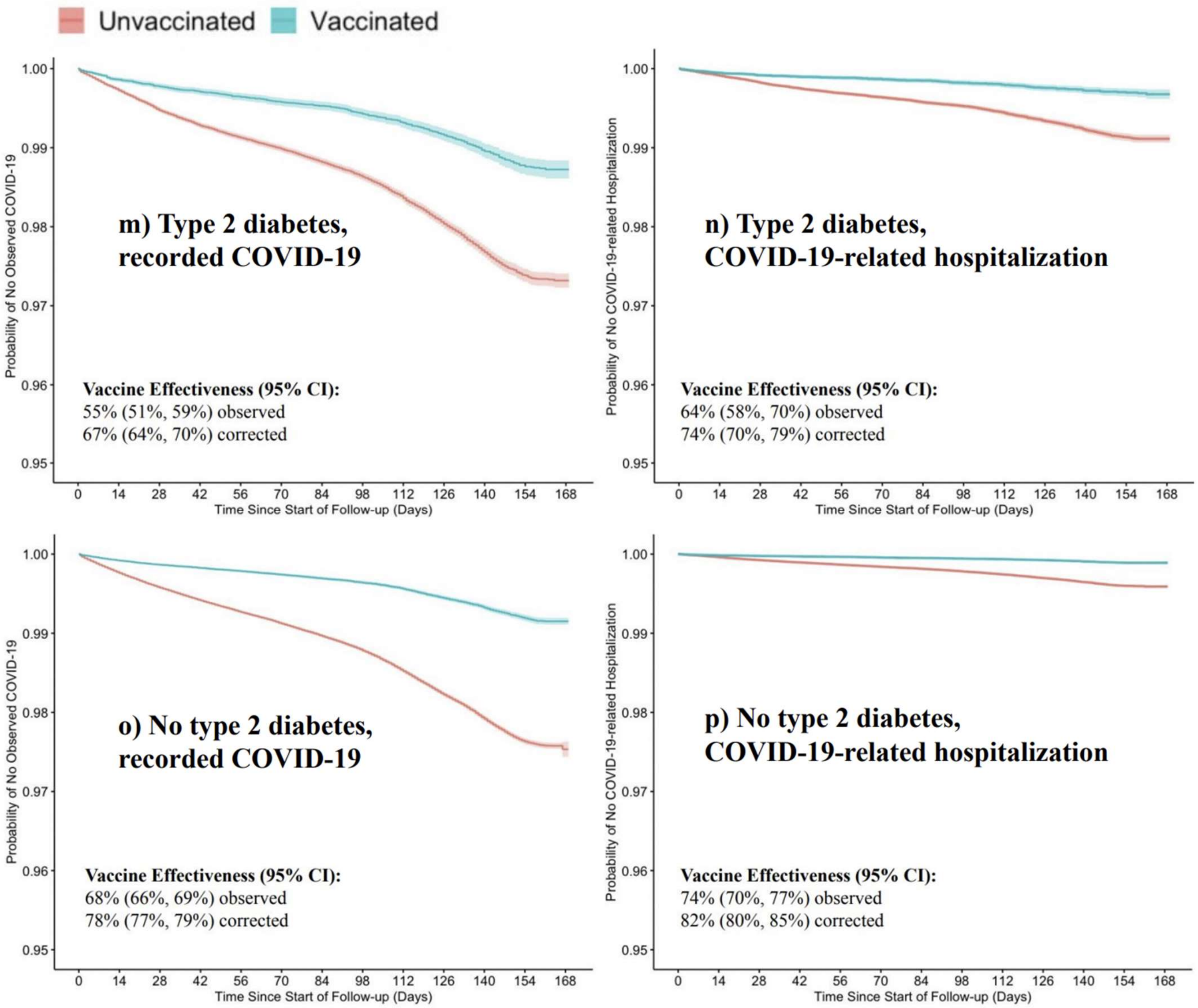

Figure S4: Follow-up day 0 is equivalent to 14 days post-index, as follow-up for outcomes begins 14 days after vaccination or matched index date. Kaplan-Meier plots include 95% confidence intervals around the survival function.

Corrected VE estimates are adjusted for under-recording of vaccinations in claims data using the approach described in **Suppl. S4**, assuming 40% under-recording of vaccinations in claims data. Note Kaplan-Meier survival curves are based on uncorrected (observed) data.

© 2022 Polinski JM et al. *JAMA Network Open.*

eFigure 5. Month-by-Month VE Plots for Subgroups Within National Cohort

a) Monthly VE estimates for recorded COVID-19 infection among age <65 subgroup

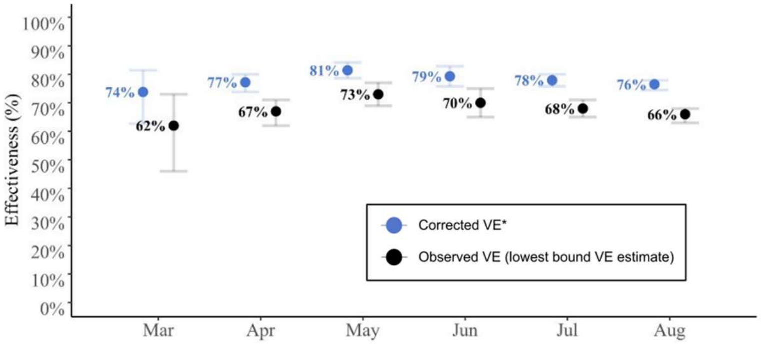

b) Monthly VE estimates for COVID-19-related hospitalization among age <65 subgroup

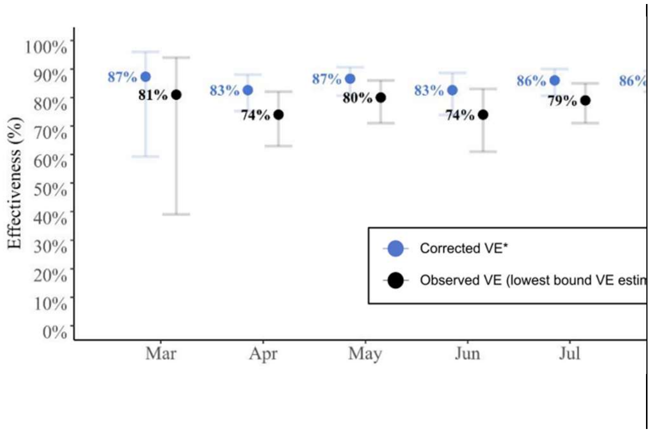

c) Monthly VE estimates for recorded COVID-19 infection among age 65+ subgroup

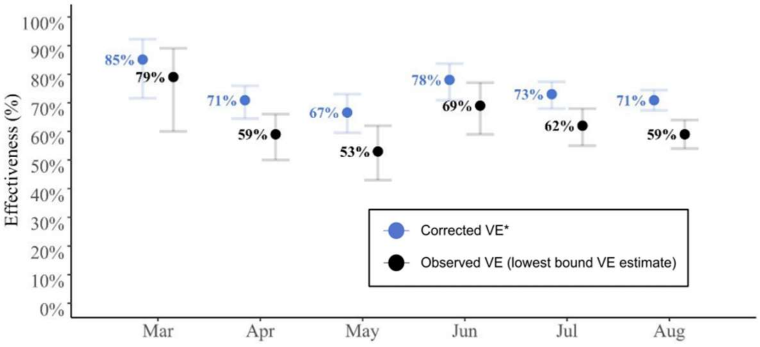

d) Monthly VE estimates for COVID-19-related hospitalization among age 65+ subgroup

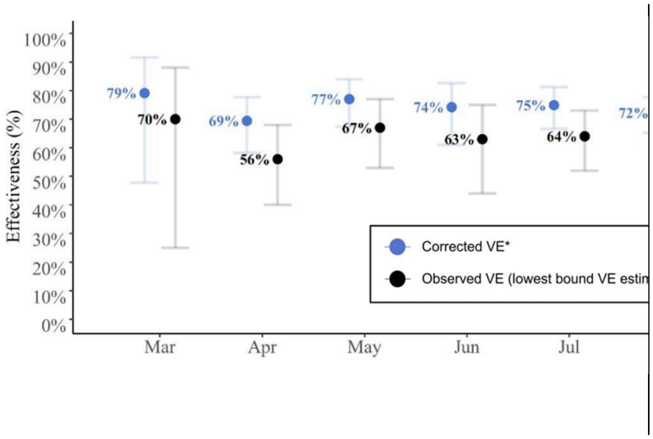

e) Monthly VE estimates for recorded COVID-19 infection among immunocompromised subgroup

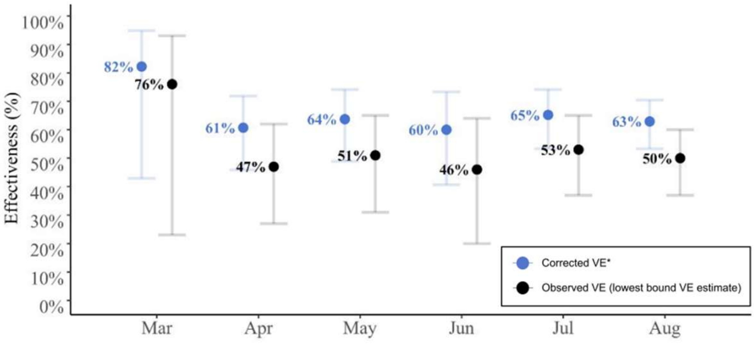

f) Monthly VE estimates for COVID-19-related hospitalization among immunocompromised subgroup

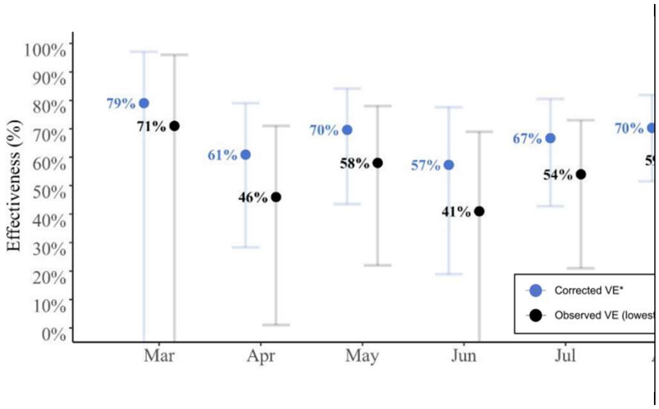

© 2022 Polinski JM et al. *JAMA Network Open.*

eFigure 5, continued.

g) Monthly VE estimates for recorded COVID-19 infection among not immunocompromised subgroup

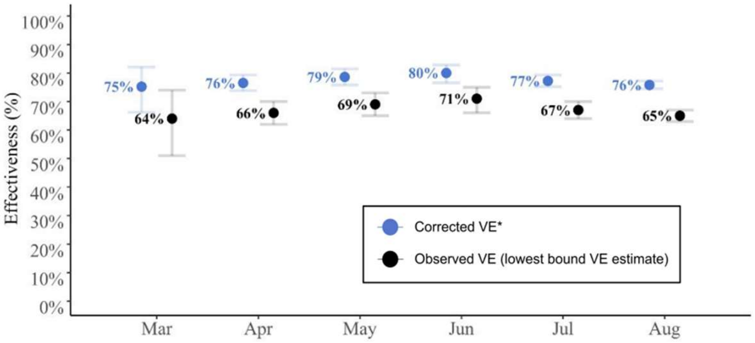

h) Monthly VE estimates for COVID-19-related hospitalization among not immunocompromised subgroup

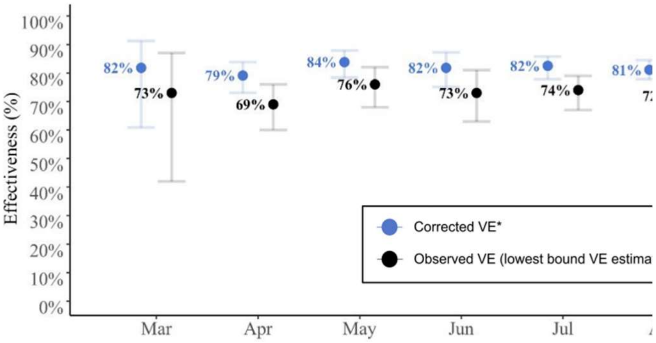

i) Monthly VE estimates for recorded COVID-19 infection among type 2 diabetes subgroup

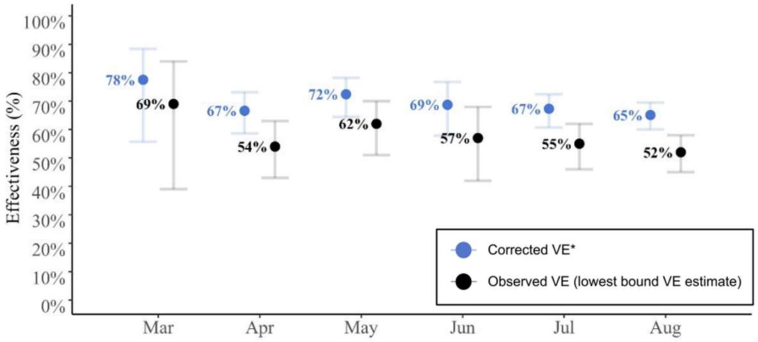

j) Monthly VE estimates for COVID-19-related hospitalization among type 2 diabetes subgroup

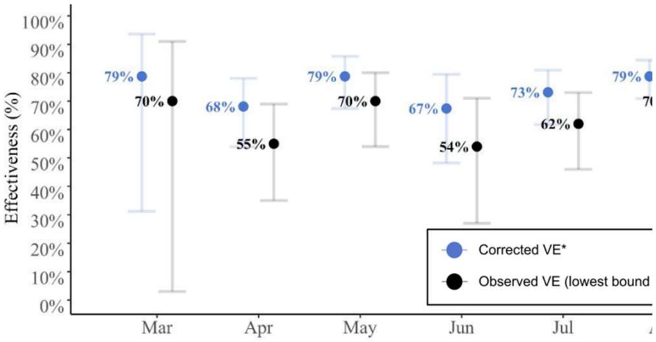

k) Monthly VE estimates for recorded COVID-19 infection among no type 2 diabetes subgroup

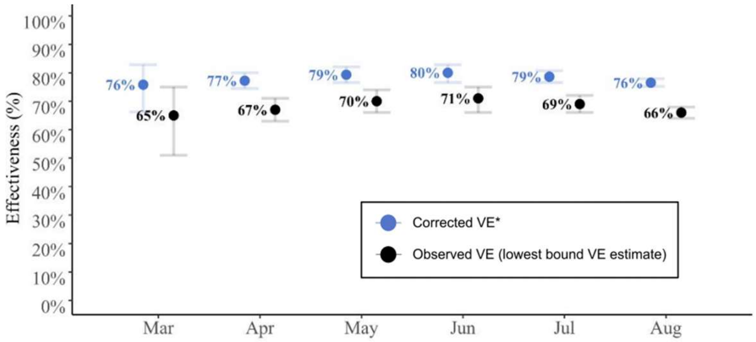

l) Monthly VE estimates for COVID-19-related hospitalization among no type 2 diabetes subgroup

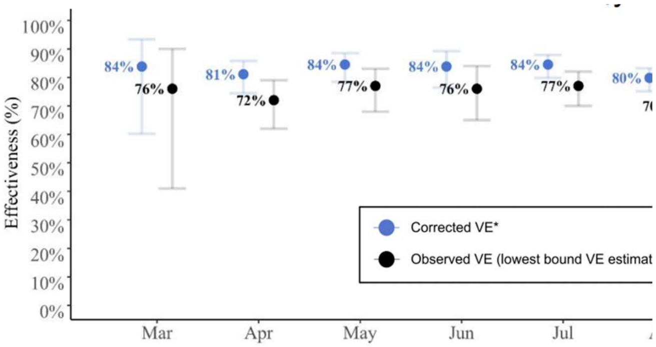

Fig S5: Insufficient sample for month-by-month VE estimates within HIV subgroups.
*Corrected VE estimates are corrected for under-recording as described in Suppl. S4, assuming 40% under-recording of vaccination in claims data.

© 2022 Polinski JM et al. *JAMA Network Open.*

eFigure 6. Plot of Schoenfeld Residuals for Subgroups Within National Cohort

a) **Age <65, recorded COVID-19 infection**

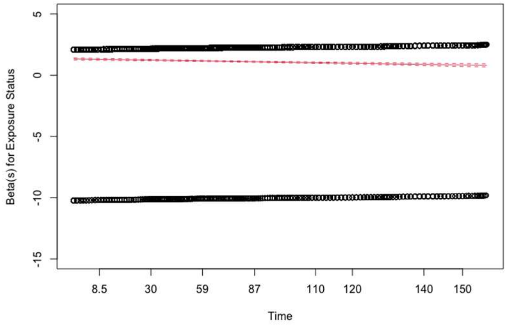

b) **Age <65, COVID-19-related hospitalization**

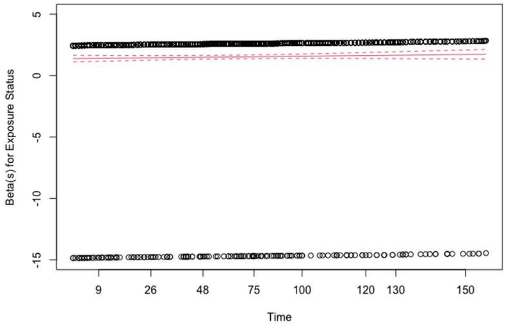

c) **Age 65+, recorded COVID-19 infection**

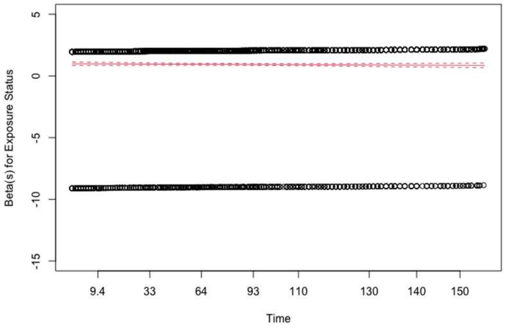

d) **Age 65+, COVID-19-related hospitalization**

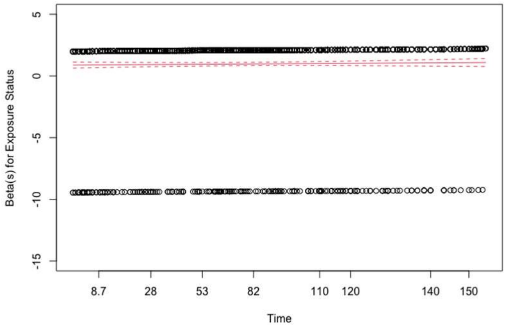

e) **Immunocompromised, recorded COVID-19 infection**

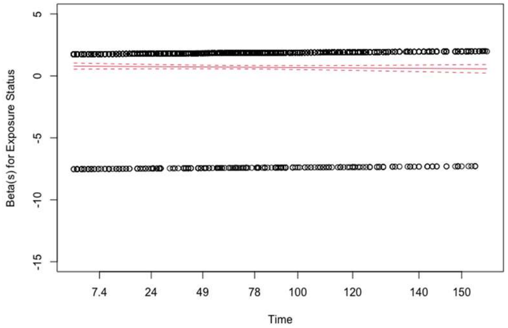

f) **Immunocompromised, COVID-19-related hospitalization**

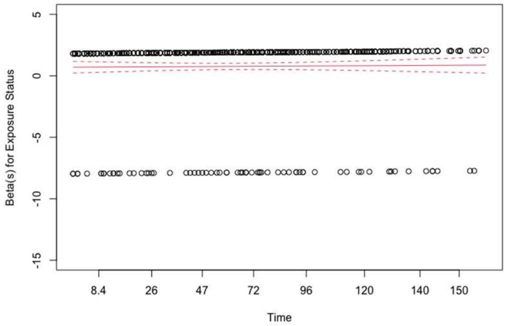

g) **Not immunocompromised, recorded COVID-19 infection**

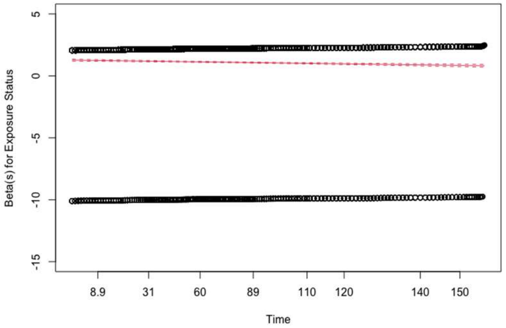

h) **Not immunocompromised, COVID-19-related hospitalization**

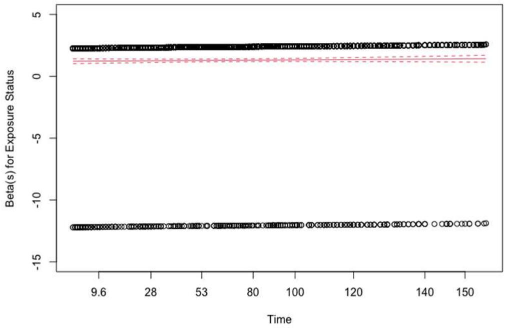

© 2022 Polinski JM et al. *JAMA Network Open.*

eFigure 6, continued.

i) HIV, recorded COVID-19 infection

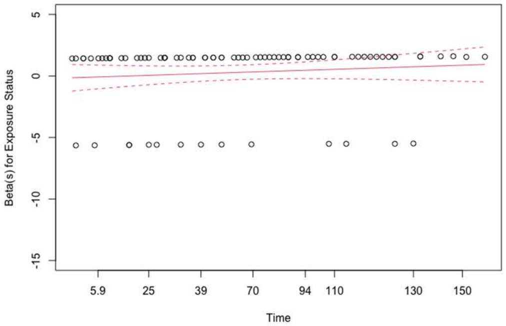

j) HIV, COVID-19-related hospitalization

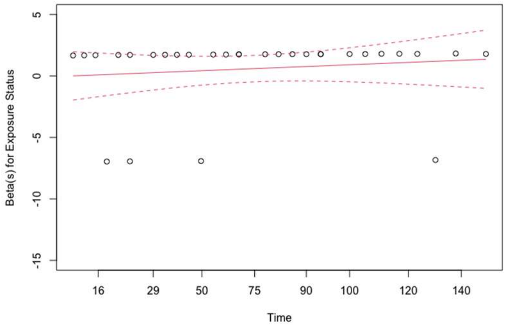

k) No HIV, recorded COVID-19 infection

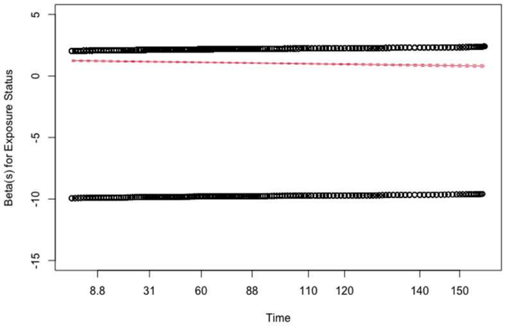

l) No HIV, COVID-19-related hospitalization

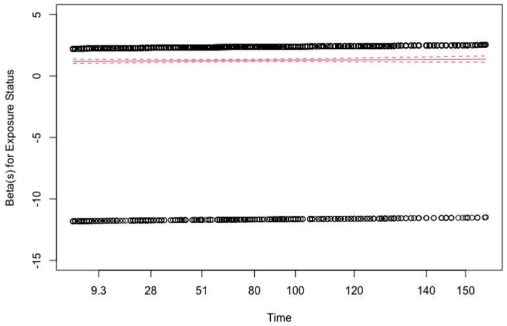

m) Type 2 diabetes, recorded COVID-19 infection

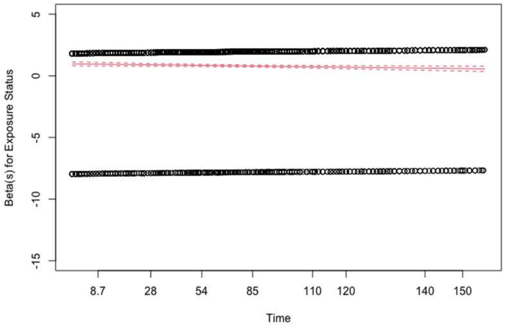

n) Type 2 diabetes, COVID-19-related hospitalization

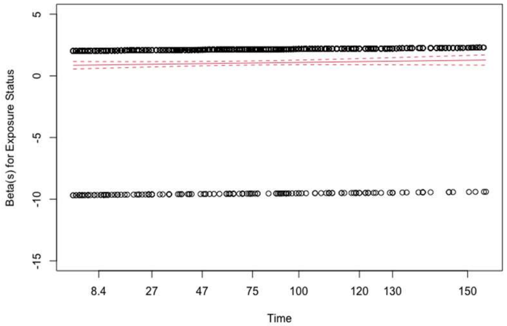

o) No type 2 diabetes, recorded COVID-19 infection

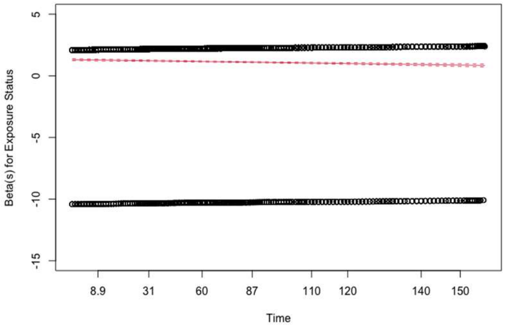

p) No type 2 diabetes, COVID-19-related hospitalization

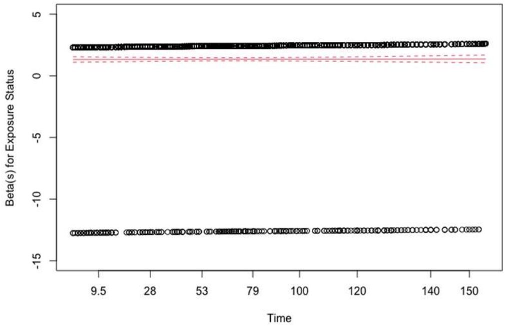

© 2022 Polinski JM et al. *JAMA Network Open.*

**eTable 1. Study Population Counts and Attrition**		
Risk-set-sampled (RSS) population:	**Count (N)**	
Individuals in dataset	168,857,729	
Individuals receiving Ad26.COV2.S vaccine from March 1, 2021 to Aug 17, 2021	4,521,812	
No record of prior Ad26.COV2.S vaccine administration	4,521,694	
Non-missing demographic information (state, 3-digit zip, age, sex)	3,975,041	
Age >=18	3,954,579	
Occurrence of (open) medical and (open) pharmacy claims during baseline	635,930	
No evidence of prior COVID-19	551,300	
No mRNA vaccine administered during baseline or at cohort entry	544,279	
Total Ad26.COV2.S Vaccinated (Exposed) individuals eligible for risk-set sampling	544,279	
Vaccinated (Exposed) individuals successfully matched to unvaccinated individual(s) during risk-set sampling	**430,668**	
Unvaccinated (Referent) individuals successfully matched to vaccinated individual(s) during risk-set sampling	**4,285,994**	
	**Count (N)**	
RSS + Propensity score matched populations:	**Ad26.COV2.S-Vaccinated (Exposed)**	**Unvaccinated (Referent)**
Overall after propensity score matching	422,034	1,645,397
Subgroup populations:		
By Age		
...Age <65	296,632	1,156,090
...Age >= 65	125,402	488,902
By immunocompromised status		
...Immunocompromised	29,115	114,325
...Not immunocompromised	392,919	1,530,417
By HIV status		
...HIV	1,710	6,735
...No HIV	420,324	1,638,342
By Type 2 Diabetes status		
...Type 2 Diabetes	65,342	254,739
...No Type 2 Diabetes	356,692	1,389,765
High-Delta-Incidence States	32,421	126,313

© 2022 Polinski JM et al. *JAMA Network Open.*

eTable 2. Characteristics of Ad26.COV2.S Vaccinated and Matched Unvaccinated Individuals in National Cohort, by Subgroup

N (%) or mean +/- SD unless otherwise noted	Age < 65			Age >= 65			Immunocompromised			Not Immunocompromised			HIV Positive			Not HIV Positive			Type 2 Diabetes			No Type 2 Diabetes		
	Vaccinated group (Exposed) N=296,632	Unvaccinated group (Referent) N=1,156,090	ASD	Vaccinated group (Exposed) N=125,402	Unvaccinated group (Referent) N=488,902	ASD	Vaccinated group (Exposed) N=29,115	Unvaccinated group (Referent) N=114,325	ASD	Vaccinated group (Exposed) N=392,919	Unvaccinated group (Referent) N=1,530,417	ASD	Vaccinated group (Exposed) N=1,710	Unvaccinated group (Referent) N=6,735	ASD	Vaccinated group (Exposed) N=420,324	Unvaccinated group (Referent) N=1,638,342	ASD	Vaccinated group (Exposed) N=65,342	Unvaccinated group (Referent) N=254,739	ASD	Vaccinated group (Exposed) N=356,692	Unvaccinated group (Referent) N=1,389,765	ASD
Age, mean (sd)	46.49 (13.33)	46.37 (13.32)	0.009	73.96 (7.99)	73.93 (7.95)	0.004	58.66 (14.82)	58.71 (15.07)	0.003	54.35 (17.50)	54.22 (17.60)	0.007	51.01 (12.01)	51.03 (12.68)	0.002	54.66 (17.38)	54.54 (17.49)	0.007	63.00 (13.07)	63.00 (13.00)	0.000	53.12 (17.61)	53.04 (17.76)	0.005
Female sex; n (%)	166,516 (56.1%)	650,071 (56.2%)	0.002	69,921 (55.8%)	273,031 (55.8%)	0.002	17,339 (59.6%)	68,295 (59.7%)	0.004	219,098 (55.8%)	854,004 (55.8%)	0.001	405 (23.7%)	1,558 (23.1%)	0.013	236,032 (56.2%)	920,323 (56.2%)	0.000	32,141 (49.2%)	125,193 (49.1%)	0.001	204,296 (57.3%)	795,727 (57.3%)	0.000
Cerebrovascular disease; n (%)	6,718 (2.3%)	25,732 (2.2%)	0.003	8,703 (6.9%)	34,057 (7.0%)	0.001	1,730 (5.9%)	6,718 (5.9%)	0.003	13,691 (3.5%)	53,377 (3.5%)	0.000	83 (4.9%)	323 (4.8%)	0.003	15,338 (3.6%)	59,914 (3.7%)	0.000	5,514 (8.4%)	21,374 (8.4%)	0.002	9,907 (2.8%)	38,862 (2.8%)	0.001
Chronic kidney disease; n (%)	8,101 (2.7%)	31,017 (2.7%)	0.003	13,803 (11.0%)	53,488 (10.9%)	0.002	3,372 (11.6%)	13,281 (11.6%)	0.001	18,532 (4.7%)	70,818 (4.6%)	0.004	187 (10.9%)	715 (10.6%)	0.010	21,717 (5.2%)	83,453 (5.1%)	0.003	11,068 (16.9%)	42,876 (16.8%)	0.003	10,836 (3.0%)	41,653 (3.0%)	0.002
Chronic obstructive pulmonary disease; n (%)	28,207 (9.5%)	109,735 (9.5%)	0.001	16,272 (13.0%)	64,096 (13.1%)	0.004	6,004 (20.6%)	23,467 (20.5%)	0.002	38,475 (9.8%)	151,199 (9.9%)	0.003	346 (20.2%)	1,368 (20.3%)	0.002	44,133 (10.5%)	172,606 (10.5%)	0.001	12,412 (19.0%)	48,232 (18.9%)	0.002	32,067 (9.0%)	126,924 (9.1%)	0.005
Cystic fibrosis*; n (%)	24 (0.0%)	99 (0.0%)	0.001	0 (0.0%)	4 (0.0%)	0.004	2 (0.0%)	5 (0.0%)	0.003	22 (0.0%)	90 (0.0%)	0.000	0 (0.0%)	0 (0.0%)	-	24 (0.0%)	107 (0.0%)	0.001	3 (0.0%)	5 (0.0%)	0.005	21 (0.0%)	102 (0.0%)	0.002
HIV; n (%)	1,307 (0.4%)	5,145 (0.4%)	0.001	154 (0.1%)	672 (0.1%)	0.004	1,461 (5.0%)	5,677 (5.0%)	0.002	0 (0.0%)	0 (0.0%)	-	1,453 (85.0%)	5,681 (84.4%)	0.017	8 (0.0%)	32 (0.0%)	0.000	281 (0.4%)	1,106 (0.4%)	0.001	1,180 (0.3%)	5,118 (0.4%)	0.006
Hypertension; n (%)	74,157 (25.0%)	286,851 (24.8%)	0.004	59,698 (47.6%)	232,040 (47.5%)	0.003	12,930 (44.4%)	50,759 (44.4%)	0.000	120,925 (30.8%)	468,430 (30.6%)	0.004	697 (40.8%)	2,719 (40.4%)	0.008	133,158 (31.7%)	516,607 (31.5%)	0.003	47,316 (72.4%)	184,326 (72.4%)	0.001	86,539 (24.3%)	336,320 (24.2%)	0.001
Immunocompromised state from blood transplant*; n (%)	3 (0.0%)	21 (0.0%)	0.002	1 (0.0%)	8 (0.0%)	0.002	4 (0.0%)	19 (0.0%)	0.002	0 (0.0%)	0 (0.0%)	-	0 (0.0%)	0 (0.0%)	-	4 (0.0%)	25 (0.0%)	0.002	1 (0.0%)	8 (0.0%)	0.003	3 (0.0%)	23 (0.0%)	0.002
Immunocompromised state from organ transplant; n (%)	1,093 (0.4%)	4,295 (0.4%)	0.001	466 (0.4%)	2,064 (0.4%)	0.008	1,558 (5.4%)	6,279 (5.5%)	0.006	1 (0.0%)	3 (0.0%)	0.000	20 (1.2%)	77 (1.1%)	0.002	1,539 (0.4%)	6,387 (0.4%)	0.004	555 (0.8%)	2,134 (0.8%)	0.001	1,004 (0.3%)	4,123 (0.3%)	0.003
Liver disease; n (%)	13,507 (4.6%)	51,401 (4.4%)	0.005	4,463 (3.6%)	17,430 (3.6%)	0.000	2,987 (10.3%)	11,646 (10.2%)	0.002	14,983 (3.8%)	57,777 (3.8%)	0.002	254 (14.9%)	985 (14.6%)	0.006	17,716 (4.2%)	68,540 (4.2%)	0.002	5,607 (8.6%)	21,790 (8.6%)	0.001	12,363 (3.5%)	47,958 (3.5%)	0.001
Malignancies; n (%)	8,842 (3.0%)	33,971 (2.9%)	0.002	9,756 (7.8%)	38,350 (7.8%)	0.002	11,081 (38.1%)	43,465 (38.0%)	0.001	7,517 (1.9%)	29,346 (1.9%)	0.000	101 (5.9%)	388 (5.8%)	0.006	18,497 (4.4%)	71,910 (4.4%)	0.001	4,339 (6.6%)	16,912 (6.6%)	0.000	14,259 (4.0%)	55,962 (4.0%)	0.001
Moderate-to-severe asthma; n (%)	3,036 (1.0%)	11,762 (1.0%)	0.001	826 (0.7%)	3,448 (0.7%)	0.006	612 (2.1%)	2,426 (2.1%)	0.001	3,250 (0.8%)	13,061 (0.9%)	0.003	28 (1.6%)	111 (1.6%)	0.001	3,834 (0.9%)	15,095 (0.9%)	0.001	935 (1.4%)	3,625 (1.4%)	0.001	2,927 (0.8%)	11,689 (0.8%)	0.002
Neurologic conditions; n (%)	81,822 (27.6%)	315,283 (27.3%)	0.007	39,715 (31.7%)	154,847 (31.7%)	0.000	12,216 (42.0%)	47,738 (41.8%)	0.004	109,321 (27.8%)	425,245 (27.8%)	0.001	654 (38.2%)	2,569 (38.1%)	0.002	120,883 (28.8%)	470,025 (28.7%)	0.002	29,732 (45.5%)	115,955 (45.5%)	0.000	91,805 (25.7%)	357,278 (25.7%)	0.001
Obesity; n (%)	48,665 (16.4%)	187,713 (16.2%)	0.005	16,793 (13.4%)	65,659 (13.4%)	0.001	6,318 (21.7%)	24,670 (21.6%)	0.003	59,140 (15.1%)	228,511 (14.9%)	0.003	285 (16.7%)	1,103 (16.4%)	0.008	65,173 (15.5%)	252,565 (15.4%)	0.002	21,946 (33.6%)	85,224 (33.5%)	0.003	43,512 (12.2%)	169,065 (12.2%)	0.001
Pulmonary fibrosis; n (%)	989 (0.3%)	3,878 (0.3%)	0.000	1,132 (0.9%)	4,755 (1.0%)	0.007	494 (1.7%)	1,950 (1.7%)	0.001	1,627 (0.4%)	6,781 (0.4%)	0.004	15 (0.9%)	60 (0.9%)	0.001	2,106 (0.5%)	8,546 (0.5%)	0.003	642 (1.0%)	2,538 (1.0%)	0.001	1,479 (0.4%)	6,208 (0.4%)	0.005
Serious heart conditions; n (%)	18,403 (6.2%)	71,484 (6.2%)	0.001	23,455 (18.7%)	91,674 (18.8%)	0.001	4,972 (17.1%)	19,584 (17.1%)	0.001	36,886 (9.4%)	144,761 (9.5%)	0.002	240 (14.0%)	917 (13.6%)	0.012	41,618 (9.9%)	163,021 (10.0%)	0.002	16,750 (25.6%)	65,491 (25.7%)	0.002	25,108 (7.0%)	99,507 (7.2%)	0.005
Sickle-cell disease; n (%)	163 (0.1%)	724 (0.1%)	0.003	32 (0.0%)	133 (0.0%)	0.001	28 (0.1%)	114 (0.1%)	0.001	167 (0.0%)	730 (0.0%)	0.002	5 (0.3%)	22 (0.3%)	0.006	190 (0.0%)	855 (0.1%)	0.003	50 (0.1%)	203 (0.1%)	0.001	145 (0.0%)	716 (0.1%)	0.005
Thalassemia; n (%)	147 (0.0%)	557 (0.0%)	0.001	64 (0.1%)	247 (0.1%)	0.000	28 (0.1%)	104 (0.1%)	0.002	183 (0.0%)	721 (0.0%)	0.000	1 (0.1%)	4 (0.1%)	0.000	210 (0.0%)	861 (0.1%)	0.001	63 (0.1%)	257 (0.1%)	0.001	148 (0.0%)	596 (0.0%)	0.001

© 2022 Polinski JM et al. *JAMA Network Open.*

Type 1 diabetes mellitus; n (%)	3,560 (1.2%)	13,670 (1.2%)	0.002	986 (0.8%)	3,828 (0.8%)	0.000	427 (1.5%)	1,651 (1.4%)	0.002	4,119 (1.0%)	16,094 (1.1%)	0.000	20 (1.2%)	71 (1.1%)	0.011	4,526 (1.1%)	17,533 (1.1%)	0.001	2,685 (4.1%)	10,440 (4.1%)	0.001	1,861 (0.5%)	7,152 (0.5%)	0.001
Type 2 diabetes mellitus; n (%)	35,665 (12.0%)	136,786 (11.8%)	0.006	29,516 (23.5%)	114,613 (23.4%)	0.002	5,967 (20.5%)	23,394 (20.5%)	0.001	59,214 (15.1%)	227,265 (14.8%)	0.006	299 (17.5%)	1,163 (17.3%)	0.006	64,882 (15.4%)	249,349 (15.2%)	0.006	65,181 (99.8%)	254,133 (99.8%)	0.002	0 (0.0%)	0 (0.0%)	-
Gagne combined comorbidity score, mean (sd)	0.53 (1.37)	0.51 (1.34)	0.010	0.94 (1.96)	0.94 (1.95)	0.000	1.99 (2.77)	1.98 (2.86)	0.003	0.55 (1.41)	0.55 (1.37)	0.004	0.69 (2.20)	0.66 (2.20)	0.014	0.65 (1.58)	0.64 (1.56)	0.005	1.73 (2.37)	1.73 (2.40)	0.001	0.45 (1.29)	0.45 (1.27)	0.001
Count of medical claims, mean (sd)	13.45 (34.39)	13.32 (32.61)	0.004	13.55 (36.85)	13.64 (32.85)	0.003	25.77 (49.43)	25.58 (47.73)	0.004	12.57 (33.66)	12.58 (31.23)	0.000	28.08 (56.66)	27.76 (59.36)	0.006	13.42 (35.01)	13.42 (32.71)	0.000	25.53 (53.08)	25.55 (50.67)	0.000	11.27 (30.22)	11.33 (28.03)	0.002
Count of pharmacy claims, mean (sd)	17.71 (19.39)	17.41 (18.92)	0.015	18.89 (17.86)	18.84 (17.79)	0.003	29.18 (24.36)	29.09 (24.36)	0.004	17.23 (18.23)	17.08 (17.98)	0.009	27.21 (23.10)	27.21 (23.86)	0.000	18.02 (18.93)	17.84 (18.64)	0.010	28.49 (23.85)	28.29 (23.92)	0.008	16.15 (17.25)	16.01 (16.93)	0.008
Recent medical claim**; n (%)	150,778 (50.8%)	582,183 (50.4%)	0.009	63,937 (51.0%)	249,649 (51.1%)	0.002	20,497 (70.4%)	80,542 (70.5%)	0.001	194,218 (49.4%)	753,655 (49.2%)	0.004	1,121 (65.6%)	4,412 (65.5%)	0.001	213,594 (50.8%)	829,142 (50.6%)	0.004	43,967 (67.3%)	171,473 (67.3%)	0.001	170,748 (47.9%)	663,102 (47.7%)	0.003
Recent pharmacy claim**; n (%)	208,907 (70.4%)	809,753 (70.0%)	0.008	93,190 (74.3%)	361,718 (74.0%)	0.007	25,881 (88.9%)	101,726 (89.0%)	0.003	276,216 (70.3%)	1,069,250 (69.9%)	0.009	1,438 (84.1%)	5,658 (84.0%)	0.002	300,659 (71.5%)	1,166,260 (71.2%)	0.008	54,134 (82.8%)	210,811 (82.8%)	0.002	247,963 (69.5%)	960,773 (69.1%)	0.008
Index months			0.006			0.005			0.007			0.006			0.022			0.004			0.003			0.006
March 2021; n (%)	94,493 (31.9%)	366,050 (31.7%)		55,823 (44.5%)	218,512 (44.7%)		11,621 (39.9%)	45,405 (39.7%)		138,695 (35.3%)	539,262 (35.2%)		644 (37.7%)	2,536 (37.7%)		149,672 (35.6%)	581,807 (35.5%)		26,212 (40.1%)	102,013 (40.0%)		124,104 (34.8%)	484,101 (34.8%)	
April 2021; n (%)	111,582 (37.6%)	437,487 (37.8%)		37,890 (30.2%)	147,672 (30.2%)		9,587 (32.9%)	37,739 (33.0%)		139,885 (35.6%)	547,986 (35.8%)		606 (35.4%)	2,355 (35.0%)		148,866 (35.4%)	582,780 (35.6%)		21,927 (33.6%)	85,646 (33.6%)		127,545 (35.8%)	499,634 (36.0%)	
May 2021; n (%)	53,484 (18.0%)	208,971 (18.1%)		19,576 (15.6%)	75,907 (15.5%)		4,706 (16.2%)	18,522 (16.2%)		68,354 (17.4%)	265,924 (17.4%)		278 (16.3%)	1,102 (16.4%)		72,782 (17.3%)	284,021 (17.3%)		10,559 (16.2%)	41,101 (16.1%)		62,501 (17.5%)	242,269 (17.4%)	
June 2021; n (%)	21,869 (7.4%)	84,994 (7.4%)		7,221 (5.8%)	27,991 (5.7%)		1,949 (6.7%)	7,731 (6.8%)		27,141 (6.9%)	104,687 (6.8%)		119 (7.0%)	494 (7.3%)		28,971 (6.9%)	112,487 (6.9%)		4,289 (6.6%)	16,819 (6.6%)		24,801 (7.0%)	95,772 (6.9%)	
July 2021; n (%)	10,131 (3.4%)	39,044 (3.4%)		3,543 (2.8%)	13,714 (2.8%)		896 (3.1%)	3,581 (3.1%)		12,778 (3.3%)	49,631 (3.2%)		50 (2.9%)	189 (2.8%)		13,624 (3.2%)	52,659 (3.2%)		1,703 (2.6%)	6,573 (2.6%)		11,971 (3.4%)	45,919 (3.3%)	
August 2021; n (%)	5,073 (1.7%)	19,544 (1.7%)		1,349 (1.1%)	5,106 (1.0%)		356 (1.2%)	1,347 (1.2%)		6,066 (1.5%)	22,927 (1.5%)		13 (0.8%)	59 (0.9%)		6,409 (1.5%)	24,588 (1.5%)		652 (1.0%)	2,587 (1.0%)		5,770 (1.6%)	22,070 (1.6%)	
U.S. Region			0.003			0.002			0.002			0.002			0.021			0.003			0.001			0.004
Northeast; n (%)	39,394 (13.3%)	153,896 (13.3%)		17,261 (13.8%)	67,125 (13.7%)		4,184 (14.4%)	16,378 (14.3%)		52,471 (13.4%)	203,661 (13.3%)		287 (16.8%)	1,117 (16.6%)		56,368 (13.4%)	218,963 (13.4%)		8,408 (12.9%)	32,755 (12.9%)		48,247 (13.5%)	187,296 (13.5%)	
Midwest; n (%)	68,002 (22.9%)	263,713 (22.8%)		27,299 (21.8%)	106,742 (21.8%)		6,925 (23.8%)	27,126 (23.7%)		88,376 (22.5%)	343,992 (22.5%)		323 (18.9%)	1,247 (18.5%)		94,978 (22.6%)	368,705 (22.5%)		14,748 (22.6%)	57,641 (22.6%)		80,553 (22.6%)	312,556 (22.5%)	
South; n (%)	123,377 (41.6%)	482,098 (41.7%)		53,664 (42.8%)	209,411 (42.8%)		12,190 (41.9%)	47,927 (41.9%)		164,851 (42.0%)	643,091 (42.0%)		720 (42.1%)	2,817 (41.8%)		176,321 (41.9%)	689,240 (42.1%)		29,305 (44.8%)	114,164 (44.8%)		147,736 (41.4%)	578,123 (41.6%)	
West; n (%)	65,859 (22.2%)	256,383 (22.2%)		27,178 (21.7%)	105,624 (21.6%)		5,816 (20.0%)	22,894 (20.0%)		87,221 (22.2%)	339,673 (22.2%)		380 (22.2%)	1,554 (23.1%)		92,657 (22.0%)	361,434 (22.1%)		12,881 (19.7%)	50,179 (19.7%)		80,156 (22.5%)	311,790 (22.4%)	
State			0.012			0.010			0.016			0.009			0.070			0.009			0.014			0.010

Characteristics reported for population matched with risk-set sampling and propensity scores. Unless otherwise noted, demographic variables are assessed at cohort entry (index) and comorbidities and clinical utilization variables are assessed during the 1 year before cohort entry.
ASD; Absolute Standardized Difference
*Variables not included in propensity score models; ** Note baseline covariate definition for Type 2 Diabetes differs slightly from subgroup definition; ***Recent medical and pharmacy claims were defined as claims beginning during the 60 days before cohort entry.

© 2022 Polinski JM et al. *JAMA Network Open.*

eTable 3. Characteristics of Ad26.COV2.S Vaccinated and Matched Unvaccinated Individuals in High Delta-Incidence States, Overall and by Subgroup																														
	**High-Delta-incidence states (overall)**			**Age < 65**			**Age >=65**			**Immunocompromised**			**Not Immunocompromised**			**HIV Positive**			**Not HIV Positive**			**Type 2 Diabetes**			**No Type 2 Diabetes**		
N (%) or mean +/- SD unless otherwise noted	**Vaccinated group (Exposed) N=32,421**	**Unvaccinated group (Referent) N= 126,313**	**ASD**	**Vaccinated group (Exposed) N= 21,632**	**Unvaccinated group (Referent) N= 84,238**	**ASD**	**Vaccinated group (Exposed) N= 10,789**	**Unvaccinated group (Referent) N= 41,962**	**ASD**	**Vaccinated group (Exposed) N= 2,397**	**Unvaccinated group (Referent) N= 9,410**	**ASD**	**Vaccinated group (Exposed) N= 30,024**	**Unvaccinated group (Referent) N= 116,646**	**ASD**	**Vaccinated group (Exposed) N= 225**	**Unvaccinated group (Referent) N= 872**	**ASD**	**Vaccinated group (Exposed) N= 32,195**	**Unvaccinated group (Referent) N= 125,459**	**ASD**	**Vaccinated group (Exposed) N= 5,447**	**Unvaccinated group (Referent) N= 21,254**	**ASD**	**Vaccinated group (Exposed) N= 26,974**	**Unvaccinated group (Referent) N= 104,947**	**ASD**
Age, mean (sd)	56.66 (16.74)	56.46 (16.73)	0.012	47.97 (12.70)	47.86 (12.71)	0.009	74.07 (7.97)	74.04 (7.94)	0.003	59.57 (14.60)	59.69 (14.55)	0.008	56.42 (16.87)	56.23 (16.89)	0.011	52.02 (12.54)	51.74 (12.55)	0.023	56.69 (16.76)	56.41 (16.79)	0.016	63.91 (12.67)	64.07 (12.67)	0.012	55.19 (17.07)	55.05 (17.10)	0.008
Female sex; n (%)	18,411 (56.8%)	72,001 (57.0%)	0.004	12,359 (57.1%)	48,410 (57.5%)	0.007	6,052 (56.1%)	23,665 (56.4%)	0.006	1,382 (57.7%)	5,430 (57.7%)	0.001	17,029 (56.7%)	66,269 (56.8%)	0.002	66 (29.3%)	256 (29.4%)	0.001	18,344 (57.0%)	71,927 (57.3%)	0.007	2,701 (49.6%)	10,548 (49.6%)	0.001	15,710 (58.2%)	61,479 (58.6%)	0.007
Cerebrovascular disease; n (%)	1,419 (4.4%)	5,416 (4.3%)	0.004	591 (2.7%)	2,210 (2.6%)	0.007	828 (7.7%)	3,204 (7.6%)	0.001	182 (7.6%)	732 (7.8%)	0.007	1,237 (4.1%)	4,691 (4.0%)	0.005	15 (6.7%)	59 (6.8%)	0.004	1,404 (4.4%)	5,366 (4.3%)	0.004	520 (9.5%)	2,014 (9.5%)	0.002	899 (3.3%)	3,442 (3.3%)	0.003
Chronic kidney disease; n (%)	1,957 (6.0%)	7,470 (5.9%)	0.005	682 (3.2%)	2,593 (3.1%)	0.004	1,275 (11.8%)	4,857 (11.6%)	0.008	313 (13.1%)	1,270 (13.5%)	0.013	1,644 (5.5%)	6,044 (5.2%)	0.013	35 (15.6%)	126 (14.4%)	0.031	1,922 (6.0%)	7,355 (5.9%)	0.005	1,037 (19.0%)	4,025 (18.9%)	0.003	920 (3.4%)	3,522 (3.4%)	0.003
Chronic obstructive pulmonary disease; n (%)	3,537 (10.9%)	13,281 (10.5%)	0.013	2,105 (9.7%)	7,928 (9.4%)	0.011	1,432 (13.3%)	5,463 (13.0%)	0.008	526 (21.9%)	2,074 (22.0%)	0.002	3,011 (10.0%)	11,303 (9.7%)	0.011	59 (26.2%)	216 (24.8%)	0.033	3,477 (10.8%)	13,185 (10.5%)	0.009	1,088 (20.0%)	4,239 (19.9%)	0.001	2,449 (9.1%)	9,310 (8.9%)	0.007
Cystic fibrosis*; n (%)	3 (0.0%)	4 (0.0%)	0.008	3 (0.0%)	7 (0.0%)	0.005	0 (0.0%)	1 (0.0%)	0.007	0 (0.0%)	0 (0.0%)	-	3 (0.0%)	7 (0.0%)	0.004	0 (0.0%)	0 (0.0%)	-	3 (0.0%)	6 (0.0%)	0.005	2 (0.0%)	1 (0.0%)	0.022	1 (0.0%)	6 (0.0%)	0.003
HIV; n (%)	205 (0.6%)	759 (0.6%)	0.004	176 (0.8%)	678 (0.8%)	0.001	29 (0.3%)	106 (0.3%)	0.003	205 (8.6%)	817 (8.7%)	0.005	0 (0.0%)	0 (0.0%)	-	203 (90.2%)	782 (89.7%)	0.018	1 (0.0%)	2 (0.0%)	0.003	50 (0.9%)	177 (0.8%)	0.009	155 (0.6%)	617 (0.6%)	0.002
Hypertension; n (%)	11,658 (36.0%)	44,504 (35.2%)	0.015	6,224 (28.8%)	23,371 (27.7%)	0.023	5,434 (50.4%)	21,068 (50.2%)	0.003	1,146 (47.8%)	4,549 (48.3%)	0.011	10,512 (35.0%)	39,754 (34.1%)	0.020	122 (54.2%)	484 (55.5%)	0.026	11,535 (35.8%)	44,056 (35.1%)	0.015	4,112 (75.5%)	16,084 (75.7%)	0.004	7,546 (28.0%)	29,010 (27.6%)	0.007
Immunocompromised state from blood transplant*; n (%)	0 (0.0%)	1 (0.0%)	0.004	0 (0.0%)	1 (0.0%)	0.005	0 (0.0%)	0 (0.0%)	-	0 (0.0%)	1 (0.0%)	0.015	0 (0.0%)	0 (0.0%)	-	0 (0.0%)	0 (0.0%)	-	0 (0.0%)	0 (0.0%)	-	0 (0.0%)	0 (0.0%)	-	0 (0.0%)	1 (0.0%)	0.004
Immunocompromised state from organ transplant; n (%)	132 (0.4%)	516 (0.4%)	0.000	86 (0.4%)	323 (0.4%)	0.002	46 (0.4%)	187 (0.4%)	0.003	132 (5.5%)	521 (5.5%)	0.001	0 (0.0%)	0 (0.0%)	-	5 (2.2%)	19 (2.2%)	0.003	127 (0.4%)	449 (0.4%)	0.006	46 (0.8%)	176 (0.8%)	0.002	86 (0.3%)	314 (0.3%)	0.004
Liver disease; n (%)	1,298 (4.0%)	4,834 (3.8%)	0.009	919 (4.2%)	3,429 (4.1%)	0.009	379 (3.5%)	1,459 (3.5%)	0.002	243 (10.1%)	974 (10.4%)	0.007	1,055 (3.5%)	3,880 (3.3%)	0.010	40 (17.8%)	158 (18.1%)	0.009	1,258 (3.9%)	4,668 (3.7%)	0.010	447 (8.2%)	1,719 (8.1%)	0.004	851 (3.2%)	3,235 (3.1%)	0.004
Malignancies; n (%)	1,431 (4.4%)	5,436 (4.3%)	0.005	643 (3.0%)	2,453 (2.9%)	0.004	788 (7.3%)	3,061 (7.3%)	0.000	886 (37.0%)	3,494 (37.1%)	0.003	545 (1.8%)	2,113 (1.8%)	0.000	19 (8.4%)	75 (8.6%)	0.006	1,412 (4.4%)	5,429 (4.3%)	0.003	348 (6.4%)	1,338 (6.3%)	0.004	1,083 (4.0%)	4,097 (3.9%)	0.006
Moderate-to-severe asthma; n (%)	219 (0.7%)	851 (0.7%)	0.000	167 (0.8%)	656 (0.8%)	0.001	52 (0.5%)	191 (0.5%)	0.004	51 (2.1%)	199 (2.1%)	0.001	168 (0.6%)	630 (0.5%)	0.003	5 (2.2%)	19 (2.2%)	0.003	214 (0.7%)	785 (0.6%)	0.005	60 (1.1%)	218 (1.0%)	0.007	159 (0.6%)	630 (0.6%)	0.001
Neurologic conditions; n (%)	9,248 (28.5%)	35,319 (28.0%)	0.013	5,873 (27.1%)	22,036 (26.2%)	0.022	3,375 (31.3%)	13,170 (31.4%)	0.002	1,028 (42.9%)	4,027 (42.8%)	0.002	8,220 (27.4%)	31,108 (26.7%)	0.016	113 (50.2%)	423 (48.5%)	0.034	9,134 (28.4%)	34,784 (27.7%)	0.014	2,515 (46.2%)	9,746 (45.9%)	0.006	6,733 (25.0%)	25,794 (24.6%)	0.009
Obesity; n (%)	5,426 (16.7%)	20,642 (16.3%)	0.011	3,869 (17.9%)	14,655 (17.4%)	0.013	1,557 (14.4%)	6,003 (14.3%)	0.004	563 (23.5%)	2,187 (23.2%)	0.006	4,863 (16.2%)	18,177 (15.6%)	0.017	55 (24.4%)	214 (24.5%)	0.002	5,371 (16.7%)	20,334 (16.2%)	0.013	1,909 (35.0%)	7,370 (34.7%)	0.008	3,517 (13.0%)	13,548 (12.9%)	0.004
Pulmonary fibrosis; n (%)	218 (0.7%)	826 (0.7%)	0.002	93 (0.4%)	343 (0.4%)	0.004	125 (1.2%)	479 (1.1%)	0.002	52 (2.2%)	203 (2.2%)	0.001	166 (0.6%)	598 (0.5%)	0.006	3 (1.3%)	13 (1.5%)	0.013	215 (0.7%)	813 (0.6%)	0.002	90 (1.7%)	344 (1.6%)	0.003	128 (0.5%)	468 (0.4%)	0.004

© 2022 Polinski JM et al. *JAMA Network Open.*

Serious heart conditions; n (%)	4,182 (12.9%)	15,907 (12.6%)	0.009	1,838 (8.5%)	6,954 (8.3%)	0.009	2,344 (21.7%)	8,975 (21.4%)	0.008	497 (20.7%)	1,980 (21.0%)	0.008	3,685 (12.3%)	13,933 (11.9%)	0.010	50 (22.2%)	197 (22.6%)	0.009	4,132 (12.8%)	15,653 (12.5%)	0.011	1,631 (29.9%)	6,355 (29.9%)	0.001	2,551 (9.5%)	9,799 (9.3%)	0.004
Sickle-cell disease; n (%)	23 (0.1%)	111 (0.1%)	0.006	20 (0.1%)	88 (0.1%)	0.004	3 (0.0%)	10 (0.0%)	0.002	6 (0.3%)	29 (0.3%)	0.011	17 (0.1%)	70 (0.1%)	0.001	2 (0.9%)	6 (0.7%)	0.023	20 (0.1%)	85 (0.1%)	0.002	8 (0.1%)	33 (0.2%)	0.002	15 (0.1%)	81 (0.1%)	0.008
Thalassemia; n (%)	18 (0.1%)	63 (0.0%)	0.002	8 (0.0%)	30 (0.0%)	0.001	10 (0.1%)	37 (0.1%)	0.002	2 (0.1%)	8 (0.1%)	0.001	16 (0.1%)	57 (0.0%)	0.002	0 (0.0%)	0 (0.0%)	-	18 (0.1%)	73 (0.1%)	0.001	4 (0.1%)	22 (0.1%)	0.010	14 (0.1%)	48 (0.0%)	0.003
Type 1 diabetes mellitus; n (%)	353 (1.1%)	1,298 (1.0%)	0.006	256 (1.2%)	963 (1.1%)	0.004	97 (0.9%)	385 (0.9%)	0.002	33 (1.4%)	128 (1.4%)	0.001	320 (1.1%)	1,133 (1.0%)	0.009	3 (1.3%)	8 (0.9%)	0.039	350 (1.1%)	1,352 (1.1%)	0.001	231 (4.2%)	887 (4.2%)	0.003	122 (0.5%)	468 (0.4%)	0.001
Type 2 diabetes mellitus; n (%)	5,432 (16.8%)	20,689 (16.4%)	0.010	2,803 (13.0%)	10,393 (12.3%)	0.019	2,629 (24.4%)	10,069 (24.0%)	0.009	523 (21.8%)	2,088 (22.2%)	0.009	4,909 (16.4%)	18,171 (15.6%)	0.021	50 (22.2%)	193 (22.1%)	0.002	5,382 (16.7%)	20,267 (16.2%)	0.015	5,432 (99.7%)	21,203 (99.8%)	0.007	0 (0.0%)	0 (0.0%)	-
Gagne combined comorbidity score, mean (sd)	0.70 (1.70)	0.68 (1.68)	0.012	0.54 (1.46)	0.52 (1.42)	0.013	1.03 (2.07)	1.02 (2.06)	0.005	2.06 (2.89)	2.09 (2.99)	0.010	0.60 (1.52)	0.57 (1.47)	0.019	1.23 (2.76)	1.13 (2.60)	0.036	0.70 (1.69)	0.68 (1.67)	0.010	1.90 (2.51)	1.90 (2.53)	0.002	0.46 (1.37)	0.45 (1.32)	0.010
Count of medical claims, mean (sd)	11.77 (30.35)	11.56 (28.02)	0.007	11.44 (29.88)	11.28 (28.96)	0.005	12.42 (31.26)	12.48 (27.82)	0.002	22.74 (41.46)	22.86 (41.54)	0.003	10.89 (29.11)	10.48 (25.89)	0.015	32.60 (56.24)	29.46 (54.98)	0.056	11.62 (30.04)	11.42 (27.59)	0.007	21.91 (44.07)	22.06 (41.23)	0.003	9.72 (26.27)	9.55 (24.33)	0.007
Count of pharmacy claims, mean (sd)	17.57 (18.29)	17.03 (17.78)	0.030	17.54 (18.99)	17.00 (18.43)	0.029	17.61 (16.82)	17.45 (17.09)	0.010	28.40 (23.90)	28.23 (23.84)	0.007	16.70 (17.48)	16.09 (16.98)	0.035	27.84 (22.92)	27.11 (23.12)	0.032	17.49 (18.24)	16.96 (17.76)	0.030	27.20 (23.13)	26.89 (23.18)	0.013	15.62 (16.48)	15.20 (15.98)	0.026
Recent medical claim**; n (%)	15,508 (47.8%)	59,649 (47.2%)	0.012	10,149 (46.9%)	38,736 (46.0%)	0.019	5,359 (49.7%)	21,059 (50.2%)	0.010	1,601 (66.8%)	6,326 (67.2%)	0.009	13,907 (46.3%)	53,068 (45.5%)	0.017	161 (71.6%)	610 (70.0%)	0.035	15,346 (47.7%)	58,956 (47.0%)	0.013	3,498 (64.2%)	13,679 (64.4%)	0.003	12,010 (44.5%)	46,224 (44.0%)	0.010
Recent pharmacy claim**; n (%)	22,803 (70.3%)	87,857 (69.6%)	0.017	15,149 (70.0%)	58,187 (69.1%)	0.021	7,654 (70.9%)	29,681 (70.7%)	0.005	2,077 (86.6%)	8,114 (86.2%)	0.012	20,726 (69.0%)	79,522 (68.2%)	0.018	192 (85.3%)	733 (84.1%)	0.035	22,610 (70.2%)	87,283 (69.6%)	0.014	4,341 (79.7%)	16,968 (79.8%)	0.003	18,462 (68.4%)	71,056 (67.7%)	0.016
Index months			0.014			0.011			0.009			0.020			0.008			0.079			0.009			0.012			0.005
March 2021; n (%)	12,287 (37.9%)	47,798 (37.8%)		7,645 (35.3%)	29,785 (35.4%)		4,642 (43.0%)	18,151 (43.3%)		938 (39.1%)	3,662 (38.9%)		11,349 (37.8%)	44,358 (38.0%)		73 (32.4%)	281 (32.2%)		12,214 (37.9%)	47,765 (38.1%)		2,182 (40.1%)	8,534 (40.2%)		10,105 (37.5%)	39,343 (37.5%)	
April 2021; n (%)	9,950 (30.7%)	39,085 (30.9%)		6,910 (31.9%)	27,042 (32.1%)		3,040 (28.2%)	11,838 (28.2%)		725 (30.2%)	2,911 (30.9%)		9,225 (30.7%)	35,890 (30.8%)		85 (37.8%)	323 (37.0%)		9,864 (30.6%)	38,555 (30.7%)		1,677 (30.8%)	6,444 (30.3%)		8,273 (30.7%)	32,186 (30.7%)	
May 2021; n (%)	5,530 (17.1%)	21,389 (16.9%)		3,718 (17.2%)	14,584 (17.3%)		1,812 (16.8%)	6,920 (16.5%)		411 (17.1%)	1,587 (16.9%)		5,119 (17.0%)	19,824 (17.0%)		38 (16.9%)	135 (15.5%)		5,492 (17.1%)	21,430 (17.1%)		881 (16.2%)	3,486 (16.4%)		4,649 (17.2%)	18,141 (17.3%)	
June 2021; n (%)	2,393 (7.4%)	9,184 (7.3%)		1,716 (7.9%)	6,642 (7.9%)		677 (6.3%)	2,650 (6.3%)		178 (7.4%)	687 (7.3%)		2,215 (7.4%)	8,434 (7.2%)		20 (8.9%)	87 (10.0%)		2,373 (7.4%)	9,209 (7.3%)		388 (7.1%)	1,532 (7.2%)		2,005 (7.4%)	7,838 (7.5%)	
July 2021; n (%)	1,582 (4.9%)	6,194 (4.9%)		1,114 (5.1%)	4,228 (5.0%)		468 (4.3%)	1,810 (4.3%)		102 (4.3%)	382 (4.1%)		1,480 (4.9%)	5,658 (4.9%)		7 (3.1%)	35 (4.0%)		1,575 (4.9%)	5,963 (4.8%)		231 (4.2%)	923 (4.3%)		1,351 (5.0%)	5,160 (4.9%)	
August 2021; n (%)	679 (2.1%)	2,663 (2.1%)		529 (2.4%)	1,957 (2.3%)		150 (1.4%)	593 (1.4%)		43 (1.8%)	181 (1.9%)		636 (2.1%)	2,482 (2.1%)		2 (0.9%)	11 (1.3%)		677 (2.1%)	2,537 (2.0%)		88 (1.6%)	335 (1.6%)		591 (2.2%)	2,279 (2.2%)	
State			0.019			0.013			0.024			0.044			0.021			0.193			0.018			0.053			0.012
FL; n (%)	7,209 (22.2%)	28,529 (22.6%)		5,253 (24.3%)	20,694 (24.6%)		1,956 (18.1%)	7,839 (18.7%)		427 (17.8%)	1,535 (16.3%)		6,782 (22.6%)	26,894 (23.1%)		34 (15.1%)	79 (9.1%)		7,175 (22.3%)	28,197 (22.5%)		934 (17.1%)	3,561 (16.8%)		6,275 (23.3%)	24,666 (23.5%)	
LA; n (%)	15,055 (46.4%)	57,675 (45.7%)		9,176 (42.4%)	35,241 (41.8%)		5,879 (54.5%)	22,500 (53.6%)		1,186 (49.5%)	4,785 (50.9%)		13,869 (46.2%)	52,926 (45.4%)		140 (62.2%)	578 (66.3%)		14,915 (46.3%)	57,231 (45.6%)		2,874 (52.8%)	10,779 (50.7%)		12,181 (45.2%)	46,929 (44.7%)	
AR; n (%)	5,624 (17.3%)	21,834 (17.3%)		4,309 (19.9%)	16,808 (20.0%)		1,315 (12.2%)	4,999 (11.9%)		464 (19.4%)	1,785 (19.0%)		5,160 (17.2%)	19,870 (17.0%)		31 (13.8%)	141 (16.2%)		5,593 (17.4%)	21,801 (17.4%)		996 (18.3%)	4,175 (19.6%)		4,628 (17.2%)	17,906 (17.1%)	

© 2022 Polinski JM et al. *JAMA Network Open.*

MO; n (%)	4,533 (14.0%)	18,275 (14.5%)		2,894 (13.4%)	11,495 (13.6%)		1,639 (15.2%)	6,624 (15.8%)		320 (13.4%)	1,305 (13.9%)		4,213 (14.0%)	16,956 (14.5%)		20 (8.9%)	74 (8.5%)		4,512 (14.0%)	18,230 (14.5%)		643 (11.8%)	2,739 (12.9%)		3,890 (14.4%)	15,446 (14.7%)	

Characteristics reported for high-Delta-incidence States (early Delta States) population matched with risk-set sampling and propensity scores. Unless otherwise noted, demographic variables are assessed at cohort entry (index) and comorbidities and clinical utilization variables are assessed during the 1 year before cohort entry.

ASD; Absolute Standardized Difference

*Variables not included in propensity score models; ** Note baseline covariate definition for Type 2 Diabetes differs slightly from subgroup definition; ***Recent medical and pharmacy claims were defined as claims beginning during the 60 days before cohort entry.

© 2022 Polinski JM et al. *JAMA Network Open.*

eTable 4. Incidence and Vaccine Effectiveness for COVID-19 and COVID-19–Related Hospitalizations: High Delta-Incidence States, Overall and by Subgroup

	Vaccinated group			Unvaccinated group			Observed (uncorrected for vaccine under-recording)		Corrected for vaccine under-recording	
	N events	Person-years	Incidence rate	N events	Person-years	Incidence rate	**HR (95% CI)**	**VE (95% CI)**	**HR (95% CI)**	**VE (95% CI)**
**Overall high-Delta-incidence States**										
Any recorded COVID-19	372	10,691	34.80	3,466	36,564	94.79	0.36 (0.32, 0.40)	64% (60%, 68%)	0.25 (0.22, 0.28)	75% (72%, 78%)
June - Aug 2021 Only**	327	7,399	44.2	2,948	24,814	118.8	0.37 (0.33, 0.41)	63% (59%, 67%)	0.26 (0.23, 0.29)	74% (71%, 77%)
COVID-19-related hospitalization	61	10,726	5.69	718	36,910	19.45	0.29 (0.22, 0.37)	71% (63%, 78%)	0.20 (0.15, 0.25)	80% (75%, 85%)
June - Aug 2021 Only**	49	7,431	6.59	592	25,127	23.56	0.28 (0.21, 0.37)	72% (63%, 79%)	0.19 (0.14, 0.25)	81% (75%, 86%)
**Subgroups for high-Delta-incidence States**										
**Age < 65**										
Any recorded COVID-19	274	7,002	39.13	2,513	23,440	107.21	0.35 (0.31, 0.40)	65% (60%, 69%)	0.25 (0.22, 0.28)	75% (72%, 78%)
COVID-19-related hospitalization	21	7,032	2.99	382	23,699	16.12	0.18 (0.12, 0.28)	82% (72%, 88%)	0.12 (0.08, 0.19)	88% (81%, 92%)
**Age >= 65**										
Any recorded COVID-19	98	3,688	26.57	895	13,100	68.32	0.38 (0.31, 0.47)	62% (53%, 69%)	0.25 (0.21, 0.31)	75% (69%, 79%)
COVID-19-related hospitalization	40	3,694	10.83	332	13,172	25.20	0.42 (0.30, 0.59)	58% (41%, 70%)	0.28 (0.20, 0.39)	72% (61%, 80%)
**Immunocompromised**										
Any recorded COVID-19	34	795	42.76	238	2,830	84.09	0.50 (0.35, 0.72)	50% (28%, 65%)	0.42 (0.29, 0.60)	58% (40%, 71%)
COVID-19-related hospitalization	9	798	11.28	67	2,851	23.50	0.47 (0.24, 0.95)	53% (5%, 76%)	0.42 (0.22, 0.86)	58% (15%, 78%)
**Not immunocompromised**										
Any recorded COVID-19	338	9,896	34.16	3,180	33,688	94.4	0.35 (0.31, 0.39)	65% (61%, 69%)	0.24 (0.21, 0.27)	76% (73%, 79%)
COVID-19-related hospitalization	52	9,928	5.24	644	34,002	18.94	0.27 (0.20, 0.36)	73% (64%, 80%)	0.18 (0.13, 0.23)	82% (77%, 87%)
**HIV Positive**										
Any recorded COVID-19	3	73	40.94	20	262	76.39	0.55 (0.16, 1.85)	45% (-85%, 84%)	-	-
COVID-19-related hospitalization	1	73	13.63	5	265	18.90	0.71 (0.08, 6.12)	29% (-512%, 92%)	-	-
**Not HIV Positive**										

© 2022 Polinski JM et al. *JAMA Network Open.*

Any recorded COVID-19	369	10,617	34.76	3,439	36,382	94.52	0.36 (0.32, 0.40)	64% (60%, 68%)	0.25 (0.22, 0.28)	75% (72%, 78%)
COVID-19-related hospitalization	60	10,652	5.63	736	36,710	20.05	0.28 (0.21, 0.36)	72% (64%, 79%)	0.19 (0.14, 0.24)	81% (76%, 86%)
**Type 2 Diabetes**										
Any recorded COVID-19	92	1,824	50.43	594	6,316	94.05	0.52 (0.42, 0.65)	48% (35%, 58%)	0.37 (0.30, 0.46)	63% (54%, 70%)
COVID-19-related hospitalization	24	1,832	13.10	219	6,363	34.42	0.37 (0.24, 0.57)	63% (43%, 76%)	0.28 (0.18, 0.43)	72% (57%, 82%)
**No Type 2 Diabetes**										
Any recorded COVID-19	280	8,867	31.58	2,822	30,193	93.46	0.33 (0.29, 0.37)	67% (63%, 71%)	0.22 (0.20, 0.25)	78% (75%, 80%)
COVID-19-related hospitalization	37	8,894	4.16	516	30,477	16.93	0.24 (0.17, 0.33)	76% (67%, 83%)	0.15 (0.11, 0.21)	85% (79%, 89%)

Unless otherwise noted, incident rates are reported per 1,000 person-years and vaccine effectiveness (VE) estimates (observed and corrected) are calculated using hazard ratios with reported 95% confidence interval limits. Corrected VE estimates are adjusted for under-recording of vaccinations in claims data using the approach described in Suppl. S4, assuming 40% under-recording of vaccinations in claims data. Insufficient outcome counts for application of under-correction methods within the HIV positive subgroup.

* High-Delta-incidence States (early Delta states) include Arkansas, Florida, Louisiana, and Missouri.

** For June-Aug 2021 results within four states (Arkansas, Florida, Louisiana, and Missouri) with high prevalence of the Delta variant of concern, incident rate ratios (IRR) after PS matching are reported instead of hazard ratios and VE is estimated using (1-IRR)x100 for patients contributing follow-up time from June 1, 2021 through Aug 31, 2021.

eTable 5. Correction of the VE Regarding Any Recorded COVID-19 Infection With Varying Assumptions About the Level of Underrecording in Claims Data

Assumed under-recording of COVID-19 vaccination status in national claims data	0%	10%	20%	30%	40%	50%	60%	70%
Sensitivity of COVID-19 vaccine exposure	100%	90%	80%	70%	60%	50%	40%	30%
Uncorrected (observed) HR (95% CI)	0.34 (0.33, 0.36)	0.34 (0.33, 0.36)	0.34 (0.33, 0.36)	0.34 (0.33, 0.36)	0.34 (0.33, 0.36)	0.34 (0.33, 0.36)	0.34 (0.33, 0.36)	0.34 (0.33, 0.36)
Uncorrected (observed) Effectiveness (95% CI)	66% (64%, 67%)	66% (64%, 67%)	66% (64%, 67%)	66% (64%, 67%)	66% (64%, 67%)	66% (64%, 67%)	66% (64%, 67%)	66% (64%, 67%)
Corrected HR (95% CI)	0.34 (0.33, 0.36)	0.32 (0.31, 0.34)	0.29 (0.28, 0.31)	0.27 (0.26, 0.28)	0.24 (0.23, 0.25)	0.21 (0.20, 0.22)	0.17 (0.17, 0.18)	0.13 (0.13, 0.14)
Corrected Effectiveness (95% CI)	66% (64%, 67%)	68% (66%, 69%)	71% (69%, 72%)	73% (72%, 74%)	76% (75%, 77%)	79% (78%, 80%)	83% (82%, 83%)	87% (86%, 87%)
					**(Primary analysis)**			

© 2022 Polinski JM et al. *JAMA Network Open.*

eTable 6. Correction of the VE Regarding COVID-19–Related Hospitalizations With Varying Assumptions About the Level of Underrecording in Claims Data

Assumed under-recording of COVID-19 vaccination status in national claims data	0%	10%	20%	30%	40%	50%	60%	70%
Sensitivity of COVID-19 vaccine exposure	100%	90%	80%	70%	60%	50%	40%	30%
Uncorrected (observed) HR (95% CI)	0.28 (0.26, 0.31)	0.28 (0.26, 0.31)	0.28 (0.26, 0.31)	0.28 (0.26, 0.31)	0.28 (0.26, 0.31)	0.28 (0.26, 0.31)	0.28 (0.26, 0.31)	0.28 (0.26, 0.31)
Uncorrected (observed) Effectiveness (95% CI)	72% (69%, 74%)	72% (69%, 74%)	72% (69%, 74%)	72% (69%, 74%)	72% (69%, 74%)	72% (69%, 74%)	72% (69%, 74%)	72% (69%, 74%)
Corrected HR (95% CI)	0.28 (0.26, 0.31)	0.26 (0.24, 0.29)	0.24 (0.22, 0.27)	0.22 (0.20, 0.24)	0.19 (0.18, 0.22)	0.17 (0.16, 0.19)	0.14 (0.13, 0.16)	0.11 (0.10, 0.12)
Corrected Effectiveness (95% CI)	72% (69%, 74%)	74% (71%, 76%)	76% (73%, 78%)	78% (76%, 80%)	81% (78%, 82%)	83% (81%, 84%)	86% (84%, 87%)	89% (88%, 90%)
					**(Primary analysis)**			

© 2022 Polinski JM et al. *JAMA Network Open.*

eTable 7. Incidence and Vaccine Effectiveness for COVID-19 and COVID-19–Related Hospitalizations Defined by Laboratory NAAT Test Results Only

	Vaccinated group			Unvaccinated group			Observed (uncorrected for vaccine under-recording)		Corrected for vaccine under-recording	
	N events	Person-years	Incidence rate	N events	Person-years	Incidence rate	**HR (95% CI)**	**VE (95% CI)**	**HR (95% CI)**	**VE (95% CI)**
**National cohort**										
Any recorded COVID-19	383	142,064	2.70	3,630	484,495	7.49	0.36 (0.32, 0.40)	64% (60%, 68%)	0.25 (0.22, 0.28)	75% (72%, 78%)
COVID-19-related hospitalization	6	142,124	0.04	127	485,055	0.26	0.16 (0.07, 0.37)	84% (63%, 93%)	0.10 (0.04, 0.24)	90% (76%, 96%)

Unless otherwise noted, incident rates are reported per 1,000 person-years and vaccine effectiveness (VE) estimates (observed and corrected) are calculated using hazard ratios with reported 95% confidence interval limits. Corrected VE estimates are adjusted for under-recording of vaccinations in claims data using the approach described in Suppl. S4.

eTable 8. Incidence and Vaccine Effectiveness for COVID-19 and COVID-19–Related Hospitalizations Among Cohort Meeting Data Extraction Criteria Before Cohort Entry*

	Vaccinated group			Unvaccinated group			Observed (uncorrected for vaccine under-recording)		Corrected for vaccine under-recording	
	N events	Person-years	Incidence rate	N events	Person-years	Incidence rate	**HR (95% CI)**	**VE (95% CI)**	**HR (95% CI)**	**VE (95% CI)**
**National cohort**										
Any recorded COVID-19	1,585	63,258	25.06	9,635	225,770	42.68	0.59 (0.56, 0.62)	41% (38%, 44%)	0.46 (0.44, 0.48)	54% (52%, 56%)
COVID-19-related hospitalization	242	63,469	3.81	1,808	226,988	7.97	0.48 (0.42, 0.55)	52% (45%, 58%)	0.37 (0.32, 0.42)	63% (58%, 68%)

***Footnote summarizing data pull criteria:**
Patients were eligible for inclusion in the dataset if they met any of the following criteria between 12/1/2019 through most recent data collection. For eligible patients, all records were included from 12/1/2018 forward:
1. ICD-10 codes for the following diagnoses found in the medical claims or chargemaster data: Influenza-like illness, Upper respiratory, Influenza, Pneumonia, COVID-19, Cough, Shortness of Breath, Fever, ARDS, Diarrhea, Fatigue, Sputum/Hemoptysis, Hypoxia
2. The following procedures codes found in the medical claims or chargemaster data: COVID-19 NAAT lab order, COVID-19 Antibody lab order, Bevacizumab, Tocilizumab, Interferon beta-1a, Eculizumab, Siltuximab
3. The following treatments found in the pharmacy claims or chargemaster data: Oseltamivir, Chloroquine, Hydroxychloroquine, lopinavir/ritonavir, Tocilizumab, Sarilumab, Baricitinib, Baloxavir Marboxil, Emtricitabine/tenofovir disoproxil fumarate, Darunavir and cobicistat, Interferon beta-1a, Fingolimod, Eculizumab, Aliskiren, Anakinra, Angiotensin II, Emapalumab-lzsg, Lucinactant (surfaxin), Ruxolitinib, Siltuximab, Caplacizumab, Dupilumab
4. COVID-19 antibody or NAAT lab tests
5. COVID-19 vaccinated in medical/pharmacy data between 12/1/2020 through most recent data collection

Sensitivity analysis required individuals to meet data extraction criteria before sampling as exposed (vaccinated) or unexposed (unvaccinated). Note patients with confirmed COVID-19 (COVID-specific diagnosis code or positive NAAT result) before cohort entry were excluded from sensitivity cohorts.

Unless otherwise noted, incident rates are reported per 1,000 person-years and vaccine effectiveness (VE) estimates (observed and corrected) are calculated using hazard ratios with reported 95% confidence interval limits. Corrected VE estimates are adjusted for under-recording of vaccinations in claims data using the approach described in Suppl. S4.

© 2022 Polinski JM et al. *JAMA Network Open.*

eTable 9. Characteristics of Ad26.COV2.S Vaccinated and Risk-Set Sample Matched Unvaccinated Individuals in National Cohort Prior to Propensity Score Matching			
N (%) or mean +/- SD unless otherwise noted	**Vaccinated group (Exposed) N=422,034**	**Unvaccinated group (Referent) N=3,955,107**	**ASD**
Age, mean (sd)	54.65 (17.36)	54.68 (17.44)	0.002
Female sex; n (%)	236,437 (56.0%)	2,225,536 (56.3%)	0.005
Cerebrovascular disease; n (%)	15,421 (3.7%)	161,902 (4.1%)	0.023
Chronic kidney disease; n (%)	21,904 (5.2%)	205,164 (5.2%)	0.000
Chronic obstructive pulmonary disease; n (%)	44,479 (10.5%)	474,085 (12.0%)	0.046
Cystic Fibrosis; n (%)	24 (0.0%)	270 (0.0%)	0.001
HIV; n (%)	1,461 (0.3%)	21,034 (0.5%)	0.028
Hypertension; n (%)	133,855 (31.7%)	1,333,442 (33.7%)	0.043
Immunocompromised from blood transplant; n (%)	4 (0.0%)	70 (0.0%)	0.002
Immunocompromised from organ transplant; n (%)	1,559 (0.4%)	20,384 (0.5%)	0.022
Liver disease; n (%)	17,970 (4.3%)	177,696 (4.5%)	0.011
Malignancies; n (%)	18,598 (4.4%)	195,706 (4.9%)	0.026
Moderate-to-severe asthma; n (%)	3,862 (0.9%)	43,217 (1.1%)	0.018
Neurologic Conditions; n (%)	121,537 (28.8%)	1,234,407 (31.2%)	0.053
Obesity; n (%)	65,458 (15.5%)	647,973 (16.4%)	0.024
Pulmonary fibrosis; n (%)	2,121 (0.5%)	27,162 (0.7%)	0.024
Serious heart conditions; n (%)	41,858 (9.9%)	448,129 (11.3%)	0.046
Sickle-cell disease; n (%)	195 (0.0%)	3,241 (0.1%)	0.014
Thalassemia; n (%)	211 (0.0%)	2,523 (0.1%)	0.006
Type 1 diabetes mellitus; n (%)	4,546 (1.1%)	44,590 (1.1%)	0.005
Type 2 diabetes mellitus; n (%)	65,181 (15.4%)	613,886 (15.5%)	0.002
Gagne combined comorbidity score, mean (sd)	0.65 (1.58)	0.67 (1.59)	0.011
Count of medical claims, mean (sd)	13.48 (35.14)	14.58 (35.18)	0.031
Count of pharmacy claims, mean (sd)	18.06 (18.96)	18.25 (18.69)	0.010
Recent medical claim*; n (%)	214,715 (50.9%)	2,117,085 (53.5%)	0.053
Recent pharmacy claim*; n (%)	302,097 (71.6%)	2,886,379 (73.0%)	0.031
Index months			0.020
...March 2021; n (%)	150,316 (35.6%)	1,378,837 (34.9%)	-
...April 2021; n (%)	149,472 (35.4%)	1,399,296 (35.4%)	-
...May 2021; n (%)	73,060 (17.3%)	701,336 (17.7%)	-
...June 2021; n (%)	29,090 (6.9%)	283,470 (7.2%)	-
...July 2021; n (%)	13,674 (3.2%)	131,826 (3.3%)	-
...August 2021; n (%)	6,422 (1.5%)	60,342 (1.5%)	-
U.S. Region			0.009

© 2022 Polinski JM et al. *JAMA Network Open.*

...Northeast; n (%)	56,655 (13.4%)	524,497 (13.3%)	-
...Midwest; n (%)	95,301 (22.6%)	882,580 (22.3%)	-
...South; n (%)	177,041 (41.9%)	1,674,787 (42.3%)	-
...West; n (%)	93,037 (22.0%)	873,243 (22.1%)	-
State			0.019

Characteristics reported for population matched with risk-set sampling. Unless otherwise noted, demographic variables are assessed at cohort entry (index) and comorbidities and clinical utilization variables are assessed during the 1 year before cohort entry.
ASD; Absolute Standardized Difference
*Recent medical and pharmacy claims were defined as claims beginning during the 60 days before cohort entry.

© 2022 Polinski JM et al. *JAMA Network Open.*
